# Quantifying firm-level economic systemic risk from nation-wide supply networks

**DOI:** 10.1038/s41598-022-11522-z

**Published:** 2022-05-11

**Authors:** Christian Diem, András Borsos, Tobias Reisch, János Kertész, Stefan Thurner

**Affiliations:** 1 Complexity Science Hub Vienna, Josefstädter Strasse 39, 1080 Vienna, Austria; 2 Institute for Finance, Banking and Insurance, Vienna University of Economics and Business, Welthandelsplatz 1, 1020 Vienna, Austria; 3 Financial Systems Analysis, Central Bank of Hungary, Szabadság tér 9, Budapest, 1054 Hungary; 4 Department of Network and Data Science, Central European University, Quellenstrasse 51, 1100 Vienna, Austria; 5 Section for Science of Complex Systems, CeMSIIS, Medical University of Vienna, Spitalgasse 23, 1090 Vienna, Austria; 6 Santa Fe Institute, 1399 Hyde Park Road, Santa Fe, NM 85701 USA

**Keywords:** Complex networks, Computational science

## Abstract

Crises like COVID-19 exposed the fragility of highly interdependent corporate supply networks and the complex production processes depending on them. However, a quantitative assessment of individual companies’ impact on the networks’ overall production is hitherto non-existent. Based on a unique value added tax dataset, we construct the firm-level production network of an entire country at an unprecedented granularity and present a novel approach for computing the economic systemic risk (ESR) of all firms within the network. We demonstrate that 0.035% of companies have extraordinarily high ESR, impacting about 23% of the national economic production should any of them default. Firm size cannot explain the ESR of individual companies; their position in the production networks matters substantially. A reliable assessment of ESR seems impossible with aggregated data traditionally used in Input-Output Economics. Our findings indicate that ESR of some extremely risky companies can be reduced by introducing supply chain redundancies and changes in the network topology.

## Introduction

Increasing the efficiency of production processes and corporate supply chains has been a dominating economic paradigm of the past decades. Popular managerial concepts that exemplify this view are reflected in keywords such as supply chain management, lean production^1^, just-in-time delivery^2^, out- and global sourcing^3–5^, or supply base reduction^6,7^. Efficiency gains are usually achieved by reducing inventory buffers, shorter lead times, supplier integration, or reducing the number of direct suppliers. Actions like these reduce production costs and increase profits. However, these actions also do have consequences in terms of resilience of the overall economy. It has been argued that increased levels of efficiency go hand in hand with a reduction of resilience^7,8^.

Supply chains of firms and consequently their production processes are highly interdependent. Supplier-buyer relations between companies lead to so-called production networks. The ongoing transformation of production networks towards higher efficiency has made these networks more vulnerable to shocks^8^. On regional scales, hurricane Katrina or the Japanese earthquake in 2011 have shown the economic impacts that can arise due to subsequent cascading shock propagation along corporate supply chains^9–11^. The COVID-19 pandemic impressively revealed that not only overall economic activity can be affected by interruptions of supply chains, but they also may lead to shortages in basic supplies^12^, affecting people directly. This became apparent, for example, in food production^13^, in vaccine supply^14^, computer chips manufacturing, and car manufacturing^15,16^.

The propagation of shocks through an economy is tightly related to classical input-output economics^17^, where, however, only industry sectors are studied. For details and relations to this work, see Supplementary Section S1. The importance of firm-level supply chains has been considered by economists, in particular the consequences of individual company failure on the overall economy^18–20^. The supply chain and production management literature studied firm level supply networks^21^ and the spreading of disruptions along supply chains under various names, such as *supply chain resilience*^8^, *snowball effect*^22^, the *ripple effect*^23^, or *nexus suppliers*^24^. Despite all these efforts, reliable and systematic estimates of firm level systemic risk for entire economies are hitherto not available.

When compared to recent progress in the assessment of systemic risk in financial networks^25,26^, the quantification of *economic systemic risk* (ESR) of individual companies in production networks is still in its infancy. From initial demonstrations of the relevance of network structure in the context of financial systemic risk^27–29^, by now it is possible to assign systemic risk to individual players in financial networks^30,31^, individual transactions^32^, and on multiple network layers^26,33^. These developments allow for novel policy paradigms for systemic risk regulation^34^. To reliably assess systemic risks detailed and correct information on the underlying networks is essential^35^. For more information on financial systemic risk and its relation to the topic of the present paper, see Supplementary Section S2.

**Figure 1:**
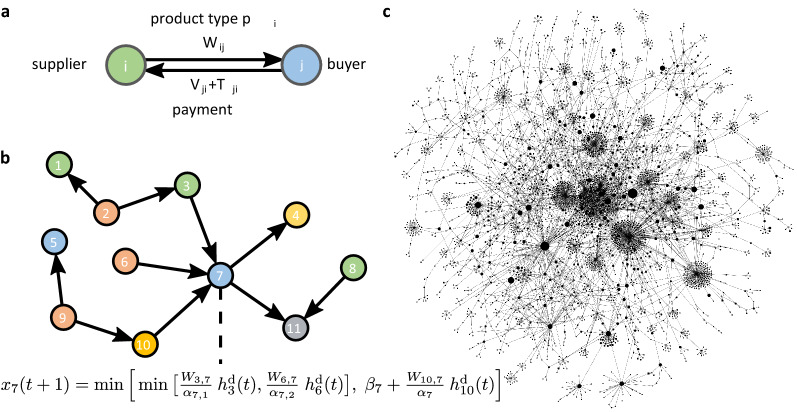
Reconstructing the production network. (**a**) Schematic supplier-buyer transaction between two companies, *i* and *j*. Supplier *i* produces products of type, $p_i$, and delivers the quantity $W_{ij}$ to buyer *j*. The buyer makes a gross payment consisting of the net price, $V_{ji}$, and the value added tax, $T_{ji}$. (**b**) Production network consisting of 11 firms. Color represents firms’ industry affiliation, $p_i$. Delivered goods and services are recorded in the weighted adjacency matrix, $W_{ij}$; element $W_{ij}$ records the volume delivered from *i* to *j*, and $h^\text {d}_i(t)$ represents the relative production level of firm *i* at time *t*. The generalized Leontief production function from Eq. (1) is shown for firm 7 as an example. It uses three types of input, $p_3, p_6$ and $p_{10}$ from firms 3, 6, and 10 and can produce the amount $x_7(t+1)$ of product $p_7$. Note, firms 3 and 6 supply essential products to firm 7, whereas firm 10 sells non-essential goods to firm 7. Firm 7 sells its output to firms 4 and 11. For simplicity we omit edge weights. (**c**) Section of the Hungarian production network with 4,070 nodes and 4,845 links. Node size corresponds to the total strength (proxy for company size). Many supply “chains” are connected and form a tightly knit supply network.

It is impossible for decision makers to manage economic systemic risks proactively without understanding which companies pose exceptionally high risk to the entire economy in case of their (temporary) failure. It is therefore important to develop methodology to quantify these risks. Here we show how firm level supply transaction data can be used to compute an *economic systemic risk index* (ESRI) for *every* company within an entire country.

The most important ingredient for developing a meaningful ESRI is the underlying firm level production network, consisting of the supply relations between companies and their production processes. Country-wide production networks can be reconstructed from firm level value added tax (VAT) transaction data^36,37^. The value added tax exists in all OECD countries, except for the USA^38^. The construction of the supply network is schematically depicted in Fig. 1a. For every sales transaction between a supplier *i* and buyer *j*, the monetary value of the goods and services sold, $V_{ji}$, can be inferred, from the tax rate, $\tau$, and the tax amount paid, $T_{ji}= \tau V_{ji}$. We use this as an estimate for the volume, $W_{ij}$, of product type, $p_i$, delivered from supplier *i* to buyer *j*. Note that the proxy (volume = price $\times$ quantity) is a basic assumption in economics^17,39^. For notation, in-links to a node represent supply transactions (buying); out-links are sales transactions. We define the in-strength of node *i* as the sum of all its in-links, $s_i^\text {in} = \sum _{j=1}^{n} W_{ji}$ (volume of purchased products), the out-strength is the sum of all out-links, $s_i^\text {out} = \sum _{j=1}^{n} W_{ij}$ (sales). The (total) strength is defined as $s_i=s_i^\text {in}+s_i^\text {out}$ and serves as a proxy for firm-size.

To assess the importance of the supply transaction, $W_{ij}$, between firms *i* and *j*, it is essential to know which product type, $p_i$, is exchanged and how it is used in the production process of firm *j*. We use the term “products” for goods *and* services. Economists typically resort to the simplifying assumption that every company produces one out of *m* different products that is determined by the company’s industry classification. We use the industry affiliation vector, *p*, to assign one of *m* industries to each firm *i*, $p_i \in \{1, 2, \dots , m \}$. We use the NACE (Statistical Classification of Economic Activities in the European Community ^40^) classification scheme on the 4-digit level with $m=615$ categories. For more details on industry classifications, see Supplementary Section S3.

The production process of a company is commonly described with a *production function*^41^, $x_i = f_i(\Pi _i, l_i, c_i)$, that determines the (maximal) amount of output, $x_i$, firm *i* can produce of product type, $p_i$, with a given amount of intermediate products, $\Pi _i=(\Pi _{i1}, \Pi _{i2}, \dots \Pi _{im})$, its employees (labour), $l_i$, and manufacturing equipment (capital), $c_i$. The amount of input *k*, firm *i* uses for its production, $\Pi _{ik}$, can be mapped to the supply network via the in-links to its suppliers, $W_{ji}$, by the relation $\Pi _{ik}=\sum _{j=1}^{n} W_{ji}\delta _{p_j,k}$, where $\delta _{a,b}$ is the Kronecker delta. Therefore, a production function, *f*, allows us to determine how much firm *i* can still produce if a supplier *j* fails to deliver its products of type, $p_j=k$, to firm *i* and hence reduces the available amount, $\Pi _{ik}$, of input *k*. Fig. 1b illustrates this with a small example production network. By matching every supply link, $W_{ji}$ to the respective input, $\Pi _{ik}$ in the production function, we can see how the production of a given firm depends on the current state of the network. As an example, we show the generalized Leontief production function (see Eq. (1)) for firm 7. It uses three different types of inputs, $p_3, p_6$ and $p_{10}$, supplied by firms 3, 6, and 10 and produces the amount $x_7(t+1)$ of product $p_7$. Firm 7 sells its output to firms 4 and 11. For simplicity we omit edge weights. Should one of the in-links diminish or vanish, this impacts the output of firm 7. This illustrates the *downstream* shock propagation of a *supply shock*. Vice versa, if 4 or 11 decide to no longer buy from 7, the in-links $W_{3,7},W_{6,7}, W_{10,7}$ would be no longer needed. This illustrates the *upstream* propagation of a *demand shock*. Figure 1 illustrates these shock propagation dynamics in detail.

The specific choice of the production function, $f_i$, determines the intensity of the downstream shock propagation. Frequently used production functions include the constant elasticity of substitution (CES)^10,42,43^ and its special cases, the Cobb-Douglas^20^ and the Leontief^11,44^ production functions, see Supplementary Section S4 for details. Here we take a short term shock propagation perspective and consider a *generalized Leontief production function* (GLPF) that accounts for the fact that even for companies with physical production processes, not all procured inputs are essential in the short term. For example, in tire production, rubber intermediates are essential inputs, while consulting services are not in the short term. The GLPF treats non-essential inputs in a linear fashion, while essential inputs are treated in the non-linear Leontief way. Note that^44^ provides a study on which inputs are essential for 56 industry sectors. We denote the set of essential inputs by $\mathscr {I}_i^\text {es}$ and non-essential inputs by $\mathscr {I}_i^\text {ne}$, respectively. We define the GLPF as

1 $$\begin{aligned} x_i = \min \Bigg [ \min _{k \in \mathscr {I}_i^\text {es}} \Big [ \frac{1}{\alpha _{ik}}\Pi _{ik}\Big ], \, \beta _{i} + \frac{1}{\alpha _i} \sum _{k \in \mathscr {I}_i^\text {ne}} \Pi _{ik} , \; \frac{1}{\alpha _{l_i}}l_i, \; \frac{1}{\alpha _{c_i}}c_i \; \Bigg ] \, , \end{aligned}$$

where $\alpha _{ik}$ are technologically determined coefficients and $\beta _{i}$ is the production level that is possible without non-essential inputs $k \in \mathscr {I}_i^\text {ne}$; $\alpha _{i}$ is chosen to interpolate between the full production level (with all inputs) and $\beta _{i}$. Both, $\alpha$ and $\beta$, are determined by *W*, $\mathscr {I}_i^\text {es}$ and $\mathscr {I}_i^\text {ne}$. Note, that we assume labour, $l_i$ and capital, $c_i$, to be fixed (in the short term) and omit them in the following analysis and notation. The Leontief production function is a special case of Eq. (1) if all inputs are essential. The linear production function is the special case when all inputs are non-essential; see Supplementary Section S4. Another essential aspect that determines downstream shock propagation intensity is how easily failing suppliers can be replaced. For our purposes we assume that companies with a low market share are easier to replace, than firms with high market shares; the exact mechanism is described in the Methods Section Eq. (8) and in Supplementary Section S 5.

**Figure 2:**
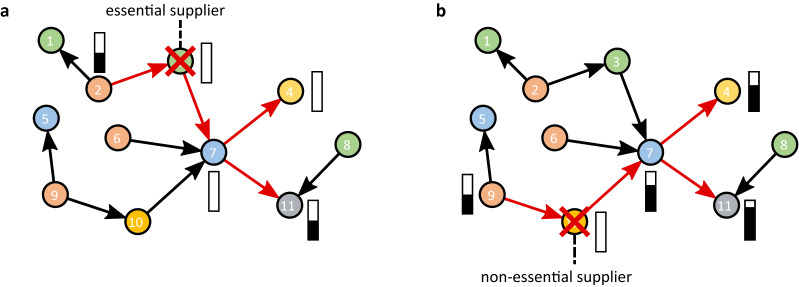
Schematic example of downstream and upstream shock propagation with generalized Leontief production functions. Red links propagated shocks; bars next to affected firms denote the resulting production level. Supplier replaceability and edge weights are omitted for simplicity. (**a**) Simulated production losses due to the failure of firm 3, $h_{3}^\text {d}(1)=h_{3}^\text {u}(1)=0$ (red cross). Firm 3 is an essential supplier to firm 7, i.e. firm 7 can not produce without its supplies, $h_7^\text {d}(2)=0$, and forwards the shock further downstream to firms 4, $h_4^\text {d}(3)=0$, and 11, $h_{11}^\text {d}(3)=1/2$. Note that firm 7 supplies a for firm 11 non-essential input. In the upstream direction firm 2 suffers a 50% demand shock, $h^\text {u}_2(2)=1/2$. (**b**) Simulated production losses due to the failure of firm 10 (red cross), $h_{10}^\text {d}(1)=h_{10}^\text {u}(1)=0$. Firm 10 supplies non-essential inputs to firm 7, i.e. firm 7 has to reduce only 1/3 of its production to $h_7^\text {d}(2)=2/3$, hence, firms 4 and 11 must reduce production to $h_{4}^\text {d}(3)=2/3$ and $h_{11}^\text {d}(3)=5/6$. Upstream, firm 9 must reduce production to $h^\text {u}_9(2)=1/2$.

### Quantifying the economic systemic risk of firms

Given the production network represented by the supply network, *W*, the industry vector, *p*, and the production functions, *f*, we can quantify the economic systemic risk (ESR) of any firm $j'$ in the following way. We estimate the production losses caused by the hypothetical failure of firm $j'$, by simulating the *direct* and *indirect* upstream and downstream effects of $j'$ not demanding any products from its suppliers and not supplying any products to its customers. Our proposed simulation takes the following steps.

First, we define the empirically observed supply network, *W*(0), (for a given year) as our initial stable state and calibrate each firms’s generalized Leontief production functions to this initial state $t=0$, as shown in Supplementary Section S4. The calibration implies that in the initial state the production output, $x_i^\text {d}(0) = f_i(\Pi _{i1}(0), \Pi _{i2}(0), \dots \Pi _{im}(0))$ that firm *i*, can produce with inputs, $\Pi _{ik}(0)$, delivered from its suppliers is equal to the sum of all its sales transactions, $x^\text {u}_i(0)=\sum _{l=1}^{n} W_{il}(0)$, to its customers, i.e. $x_i^\text {d}(0) = x^\text {u}_i(0) = x_i(0)$ for all firms *i*. We call $x^\text {d}(0)$ the downstream constrained production levels and $x^\text {u}(0)$ the upstream constrained production levels. This differentiation is necessary, because up- and downstream shocks propagate differently.

Second, we assume that firm $j'$ — for which we want to compute the ESR — stops buying from its suppliers and supplying to its buyers at time $t=1$. Consequently, we set the out-links $W_{j'i}(1)=0$, for all *i* and the in-links $W_{lj'}(1)=0$, for all *l* and $x_{j'}(1) = x_{j'}^\text {d}(1) = x_{j'}^\text {u}(1)=0$. To specify the recursive downstream and upstream update equations, we define for each firm *i* the fraction of its original production in the initial state $t=0$ it can maintain after receiving downstream and upstream shocks up to time *t* as $h_i^\text {d}(t)= \frac{x^\text {d}_{i}(t)}{x_{i}(0)}$ and $h_i^\text {u}(t)= \frac{x^\text {u}_{i}(t)}{x_{i}(0)}$, respectively.

Third, we simulate how the supply and demand reductions propagate downstream to the direct and indirect buyers and upstream to the direct and indirect suppliers of firm $j'$, by recursively updating the production levels of all firms in the network. We compute for each firm *i* the amount it can produce at $t+1$, given the production levels of its suppliers, $h^\text {d}_j(t)$, at time *t* as

2 $$\begin{aligned} x_i^{\text {d}}(t+1) = \min \Bigg [ \min _{k \in \mathscr {I}_i^\text {es}} \left( \frac{1}{\alpha _{ik}}\sum _{j=1}^{n} W_{ji} h_j^\text {d}(t) \delta _{p_j,k}\right) , \beta _{i} + \frac{1}{\alpha _i} \sum _{k \in \mathscr {I}_i^\text {ne}} \sum _{j=1}^{n} W_{ji} h_j^\text {d}(t) \delta _{p_j,k} \Bigg ] \quad . \end{aligned}$$

Then, the amount firm *i* produces at $t+1$, given the production level of its buyers, $h^\text {u}_l(t)$, at time *t* is computed as

3 $$\begin{aligned} x_i^{\text {u}}(t+1) = \sum _{l=1}^{n} W_{il}h_l^\text {u}(t) \quad . \end{aligned}$$

Note, that this implies that companies do not produce simply on stock if there is no demand. Furthermore, setting $W_{ij}(t)$ to $W_{ij}(0)\cdot h^\text {d}_i(t)$ in Eq. (2) implies that if firm *i* receives a downstream shock, it will forward it to it’s customers, *j*, *pro rata*, i.e., according to their initial sales shares. This is the commonly assumed *proportional rationing* mechanism. Similarly, we assume in Eq. (3) a proportional rationing for upstream shocks, by setting $W_{li}(t)$ to $W_{li}(0)\cdot h^\text {u}_i(t)$.

The failure of firm $j'$ — i.e. $h^\text {u}_{j'}(1)=h^\text {d}_{j'}(1)=0$ — is propagated through the network by iterating the dynamics captured by Eqs. (2) and (3) until the network reaches a new stable state at time *T*. The stable state is reached at time $T = \min _t\{t \in \mathbb {N} \;| \max \big [ h^d(t)-h^d(t+1) , \; h^u(t)-h^u(t+1) \big ] \; \le \epsilon \} +1$, where $\epsilon =10^{-2}$ is chosen as a convergence threshold. Therefore, we assume that once all shocks are smaller than $\epsilon$, the network dynamics stop — i.e. the production level of the firm is no longer adapted, when shocks are becoming smaller than $\epsilon$ — and the corresponding time point of convergence is *T*. Note that in this context *t* can not be interpreted as real time steps, but are the iteration steps of the recursion. The final production level of every firm *i* is set to $h_i(T)=\min (h_i^d(T),h^u_i(T))$, i.e. we assume that each firm produces what it can produce with the available inputs from its suppliers, but not more than what it can sell to its buyers at time *T*. Note that Eqs. (2) and (3) are iterated independently of each other, i.e. if a firm receives upstream and downstream shocks during the simulation, we ignore a potential interaction of the shocks. However, in practice these shocks can interact, e.g., a firm reducing its production due to a lack of one key input will reduce its purchases from suppliers of other inputs (horizontal contagion).

Finally, we compute the economic systemic risk index, ESRI$_{j'}$, of firm $j'$ by summing up the output reduction of each firm *i* in the production network as

4 $$\begin{aligned} \mathrm{ESRI}_{j'} = \sum _{i=1}^{n} \frac{s^\text {out}_i }{\sum _{l=1}^n s^\text {out}_l }\big (1-h_i(T) \big ) \quad . \end{aligned}$$

The quantity $\mathrm{ESRI}_i$ can be interpreted as fraction of the overall output in the production network that is likely to be affected if firm $j'$ (temporarily) fails. The full algorithm is described in the Methods Section S5.

We illustrate the downstream and upstream shock propagation mechanisms for the GL production function based on the example network in Fig. 1b. Figure 2a depicts the inital failure of firm 3, an essential supplier to firm 7. Figure 2b depicts the initial failure of firm 10, a non-essential supplier to firm 7. It is clearly visible that essential supplier causes considerably more production losses downstream, but both cause the same upstream production losses.

For the empirical analysis we calibrate the GL in Eq. (1) to four scenarios based on the firms’ NACE classifications. First, in a hypothetical purely linear production scenario (LIN), all firms have linear production functions, i.e. only non-essential inputs, and $\mathscr {I}_i^\text {ne} = \{\text {A01},\dots , \text {U99}\}$). Second, in a purely Leontief scenario (LEO), all firms have Leontief functions, i.e. only essential inputs, $\mathscr {I}_i^\text {es} = \{\text {A01},\dots , \text {U99}\}$. Third, in a mixed scenario (MIX) we assume all firms within NACE classes A01-F43 (physical production) have only essential inputs $\mathscr {I}_i^\text {es} = \{\text {A01},\dots , \text {U99}\}$ and all firms within NACE classes G45-U99 (non-physical production) have only non-essential inputs $\mathscr {I}_i^\text {ne} = \{\text {A01},\dots , \text {U99}\}$. Fourth, in the generalized Leontief scenario (GL) we assume that for firms within NACE A01-F43, the set of essential inputs consists of $\mathscr {I}_i^\text {es} = \{\text {A01},\dots , \text {F43}\}$ (supplied by physical producers) and the set of non-essential inputs consists of $\mathscr {I}_i^\text {ne} = \{\text {G45},\dots , \text {U99}\}$ (supplied by non-physical producers), while for firms within classes NACE G45-U99 we assume they have only non-essential inputs $\mathscr {I}_i^\text {ne} = \{\text {A01},\dots , \text {U99}\}$. We assume that firms within NACE A01-F43 have a physical production process, while firms within NACE G45-U99 predominantly trade or provide services. Note that LIN and LEO provide lower and upper bound scenarios for more realistic situations, where firms have distinct types of production functions as in the MIX and GL scenarios.

## Results

The directed production network *W* is reconstructed from fully anonymized VAT micro data of the Hungarian Central Bank. We consider all links, $W_{ij}$, where at least two recurring supply transactions occurred; see Supplementary Section S7 and^37^. Figure 1c shows a section of 4,070 companies and 4,845 links of the empirically reconstructed Hungarian production network, $W_{ij}$. The section is obtained by sampling 1,500 random nodes and considering all their direct suppliers, yielding 6,113 nodes. Only the giant component (4,070 nodes) is shown. The entire network consists of $n=91,595$ companies; it is too large and dense to visualize its structure in a meaningful way. Already this small section reveals the fact that production by no means happens along independent supply *chains*, but on a tightly interwoven *complex network*. We can clearly see extended supply chains that are merged into a supply network. The network has an apparent core periphery structure. For visualizations of other aspects of the production network, see Supplementary Section S8.

**Figure 3:**
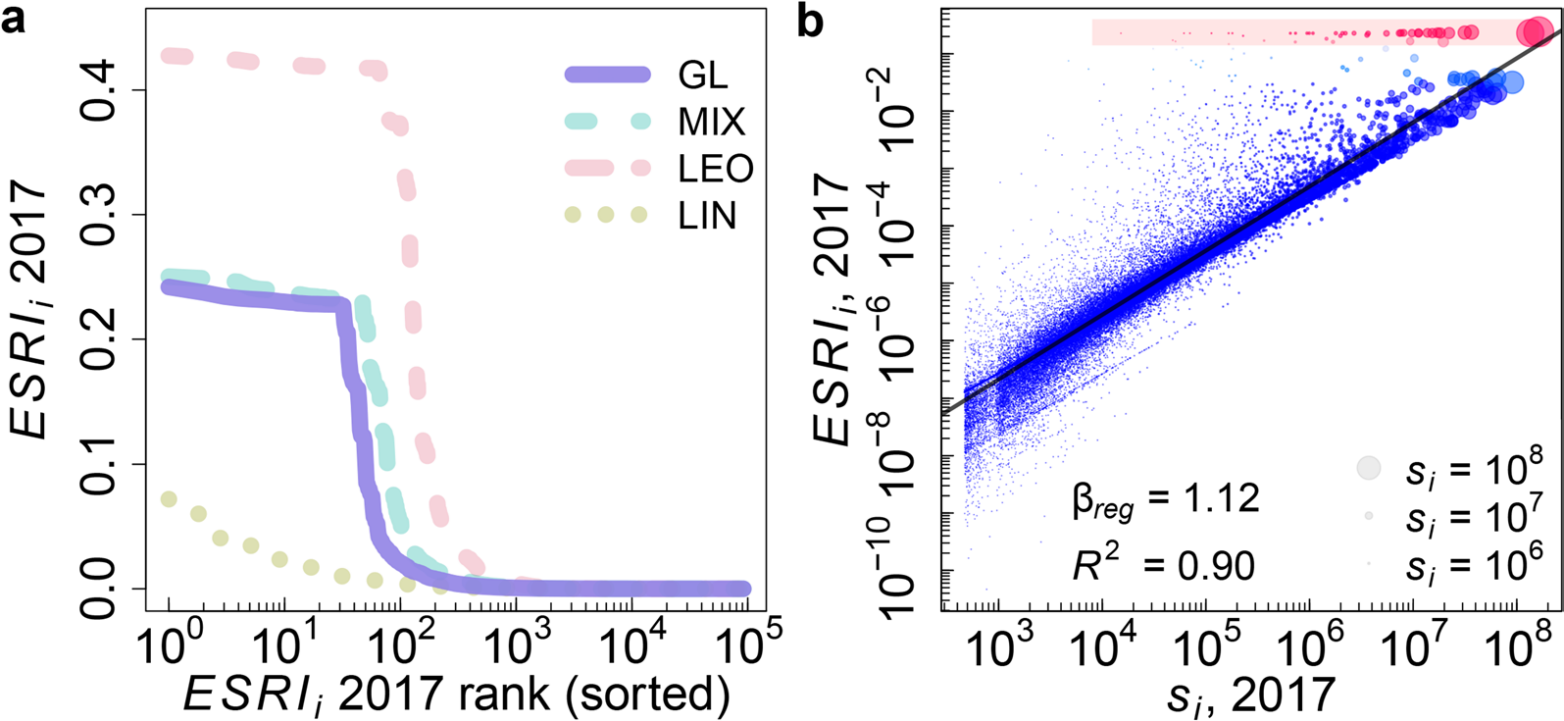
Economic systemic risk of companies. (**a**) Economic systemic risk profile (distribution) $\mathrm{ESRI}_i$ of $n=91,595$ companies in linear-log scale for 2017. Distributions are rank-ordered, meaning the most risky company is to the very left. The blue line shows the result for the realistic GL scenario (production functions according to industry classification and classifying produced goods as essential or not). A plateau exists around an ESRI of 0.23, containing 32 firms for GL. There is a steep decline to $\mathrm{ESRI}_i \sim 0.05$ from rank 33 to 63. 165 firms have an ESRI larger than 0.01. For comparison the MIX scenario (light blue) is shown (production functions according to industry classification only). As the limiting cases we show the scenarios LIN (green), where all firms have linear production functions and LEO (red), where all firms have Leontief production functions. The tail of the ESRI profile decays as an approximate power-law. (**b**) ESRI plotted against firm strength (firm-size) in log-log scale. Symbol size represents strength $s_i$, red symbols belong to the plateau, emphasised by the shaded area. We find large and small companies in the plateau, suggesting that very high ESRI is not determined by size, even though the correlation of ESRI and strength for the bulk of the companies is high.

### Distribution of economic systemic risk

We calculate the $\mathrm{ESRI}_i$ for the realistic GL and MIX scenarios as well as for the two limiting cases, LIN and LEO for all of the 91,595 companies in the Hungarian VAT micro dataset in the year 2017 and for the 85,131 firms in 2016. Rank-ordered distributions of the ESRI are shown in Fig. 3a in linear-log scale for 2017. For 2016, see Supplementary Fig. S3a in Supplementary Section S9. For our realistic main scenario GL (blue), we find that 32 companies show extremely high levels of systemic risk, all being at a value of about 0.23, meaning that about 23% of the entire economy is affected should one of these companies fail and its supply and demand is not replaced. 63 and 165 firms have an ESRI larger than 0.05 and 0.01, respectively. For respective numbers for the other scenarios, see Table S1 in Supplementary Section S10. The situation is similar for the MIX scenario, where 47 companies belong to the plateau with values around 0.23. For the (unrealistic) reference case, LEO with *all* companies of Leontief type (red), for 66 firms we find much higher systemic risk levels of about 0.42.

**Figure 4:**
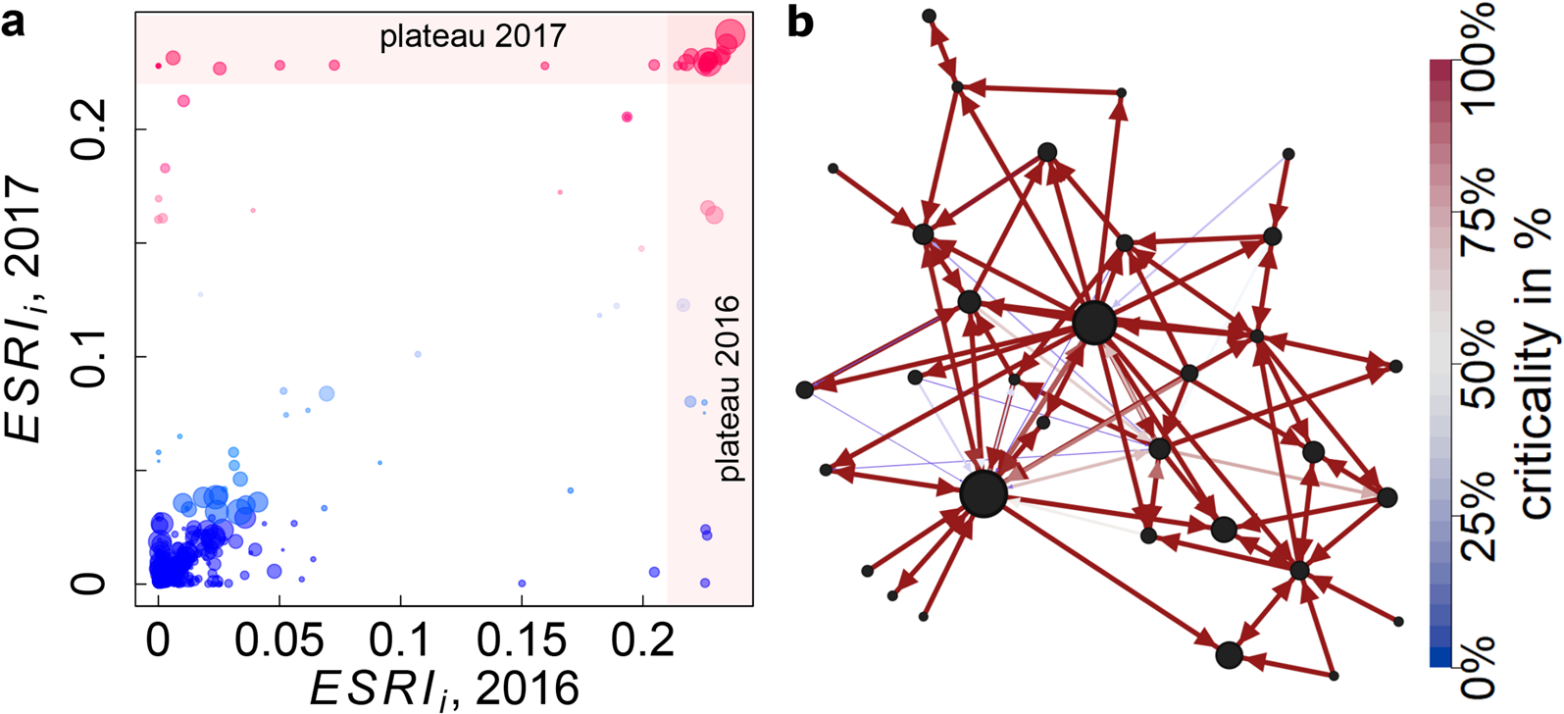
More details on ESRI. (**a**) Systemic risk in 2016 vs. 2017. Colors correspond to ESRI in 2017. Blue and red symbols indicate low and high values in 2017, respectively. Note the strong variability in the plateau firms. There is a significant correlation between ESRI in 2017 and 2016, that indicates relatively small temporal fluctuations for the bulk of the companies. (**b**) Network of the 32 most systemically risky firms (plateau) in 2017. Node size is proportional to the square root of strength. Link colors correspond to the downstream ’criticality’, i.e., the percentage of *j*’s production should *i* stop functioning, $\Lambda _{ij}^d$. Red thick (blue, thin) links indicate very large (small) losses of production. Small companies predominately supply to large high-risk companies, thereby inheriting systemic risk.

For ranks larger than a characteristic rank of 32, the pronounced plateau in the distribution is followed first by a steep decline and then by a slow decay of the ESRI values. The tail (without plateau and steep decline) can be fitted to a power-law with an exponent of roughly $\alpha ^\text {GL}=0.67$ for GL, $\alpha ^\text {MIX}=0.63$ for MIX, and $\alpha ^\text {LEO}=0.50$. For details of the fits, see Supplementary Section S10, Supplementary Fig. S4a. The shape of the rank-ordered ESRI distribution (plateau and power-law tail) is similar to the power-law relation that was found in Figure 4 in^43^. As expected, the reference case LIN (green) where *all* companies have linear production functions, generates substantially lower systemic risk levels than GL and MIX. LIN neither shows a plateau nor a power-law decay. The situation is very similar in 2016, see Supplementary Fig. S4b in Supplementary Section S10. The ESRI values presented so far, result from merging the production losses caused by the independent downstream and upstream propagation mechanisms. However, *a priori* the individual roles of the two mechanisms for economic systemic risk are still unclear. Supplementary Section S11, delineates them in detail. Figure 3a shows clearly that the high systemic risk plateaus for GL, MIX and LEO are caused by the non-linearities in production functions (downstream propagation). Figure S5a — showing the rank ordered distributions for the economic systemic risk index only computed from downstream effects, ESRI$^\text {d}_i$ — confirms that the plateaus for GL, MIX, and LEO scenarios are of the same size and height when considering only downstream effects. In the GL scenario, the ESRI$_i$ of the most risky firm would drop by 5.8% (from 0.242 to 0.228), when ignoring upstream contagion; on average the effect is negligible for the firms with ESRI$_i >0.05$. However, among firms with ESRI ranks from 64 to 165, some would be falsely deemed systemically riskless if upstream contagion was ignored. Figure S5b) — showing the rank ordered distribution for the upstream economic systemic risk index, ESRI$^\text {u}_i$ — indicates that the systemic risk solely from upstream effects can be substantial. The maximum $\mathrm{ESRI}^\text {u}$ is 0.06. 28 firms have an $\mathrm{ESRI}^\text {u}$ larger than 0.01. Figure S6 shows $\mathrm{ESRI}^\text {d}$ plotted against $\mathrm{ESRI}^\text {u}$ for GL, MIX, LEO and LIN. It becomes apparent that for all scenarios a large share of firms exhibit more upstream than downstream systemic risk. The supplier replaceability factor, $\sigma _i$, affecting only downstream systemic risk is the likely explanation for this observation. We investigate the effect of the supplier replaceability factor in more detail in Supplementary Section S12. When the replaceability factor is not taken into account, the plateaus contain more firms 8079, 1565 and 743, and the plateau levels increase to 0.53, 0.31 and 0.30, for LEO, MIX and GL, respectively. In the LIN scenario effects are small.

### Explanation for extreme systemic risk firms

To better understand which companies are forming the plateau of extremely risky companies — data protection regulations prevent us from showing company names or actual turnover — in Fig. 3b we show $\mathrm{ESRI}_i$ as a function of the strength, $s_i$ (firm size within the network). Companies on the plateau belong to industry sectors like energy, manufacturing (electrical equipment, chemicals, computer and electronics, vehicles), or repair of machinery (see Table S3 in Supplementary Section S10). Red color indicates the highly risky plateau companies; symbol size represents strength, $s_i$. Clearly, the ESRI of plateau firms (located in the shaded area) is not changing with size; in the plateau we find large and small companies (note the range of strength of 4 orders of magnitude), suggesting that firm-size is not able to predict extreme ESRI values at all. For the bulk of companies we find a strong statistically significant correlation of log-ESRI and log-strength ($R^2=0.90$ and slope $\beta _{reg}=1.12$ in log-log regression). However, for individual companies strength is not a reliable predictor of ESRI, since the spread of the ESRI extends up to 4 orders of magnitude. A more detailed regression analysis, found in Supplementary Section S13, confirms that generally firm level quantities fail to explain ESRI that is a network-based measure. Note the relation to the Hulten theorem^45^, stating — in simplified terms — that in an efficient economy the effect of a firm level shock on overall output is proportional to its revenue. Our results are in strong disagreement with this statement, adding further evidence for its limited practical validity^46^. Finally, we checked that all firms on the plateau are within NACE A01-F43, meaning that their generalized Leontief production functions contain a large share of essential inputs.

The reason for the formation of the plateau is twofold. First, about two thirds of its nodes form a strongly connected component based on highly critical supplier relationships (core of plateau). In this core the failure of one firm leads to the failure of the other members in the component, implying the default of anyone has the same consequences on the entire network (same ESRI). The second reason is that the other third of the plateau nodes are suppliers to the strongly connected component, i.e. their failure causes the failure of the nodes in the strongly connected component. These peripheral nodes inherit high ESRI values by supplying (in)directly to inherently risky companies. Note that these two features are impossible to explain with only linear production functions (LIN). To illustrate this point, in Fig. 4b, we show the network spanned by the 32 plateau firms. Node size corresponds to the square root of strength; link colors correspond to the direct down-stream impact of the supplier on the buyer node (criticality), $\Lambda ^\text {d}_{ij}$, (for the definition, see Methods Section). Red links indicate highly critical supplier-buyer relations; $\Lambda ^\text {d}_{ij}=1$ means that 100% of *j*’s production fails if *i* fails. Blue links mark non-critical supplier-buyer relations; $\Lambda ^\text {d}_{ij}=0.01$ means that *j*’s production reduces by 1% if *i* fails. It is visible that almost all links are critical for the production of the buyer.

### Changes of economic systemic risk over time

It is crucial to track the change of firms’ systemic riskiness (ESRI) over time to ensure an efficient systemic risk monitoring. To estimate the extent of the typical yearly fluctuations, we compare the ESRI values for the years 2016 and 2017 in Fig. 4b. Size is proportional to the squareroot of firm strength. Of the 68,254 firms that appear in both years, the values of ESRI in 2017 and 2016 are correlated ($\rho (\mathrm{ESRI}^{16},\mathrm{ESRI}^{17})=0.78$, $p=2.2\, 10^{-16}$). The extent of fluctuations is clearly seen in log-log representation in Supplementary Fig. S8a in Supplementary Section S14. For the logged variables we observe an even higher correlation ($\rho (\mathrm{log(ESRI)}^{16},\mathrm{log(ESRI)}^{17})=0.85$, $p=2.2\, 10^{-16}$). Supplementary Fig. S8c shows that relative changes in strength explain the relative changes in ESRI only very partially.

Figure 4a shows temporal changes of companies in the plateau between 2016 and 2017. Companies (points) in the horizontal shaded area are in the 2017 plateau, but showed (much) less ESR in 2016. Companies in the vertical shaded area are in the 2016 plateau, but not in 2017, i.e. reduced their ESRI. 20 out of 32 2016-plateau-firms remain in the 2017-plateau (intersection of shaded regions). Of the 12 companies leaving the plateau from 2016 to 2017 some remain very risky (light red) others become less risky (blue). 12 companies enter the plateau in 2017 (also firms not contained in the 2016 dataset). Possible reasons for these strong fluctuations could be that the network restructured, e.g., core plateau firms added additional suppliers for the same input, supply links were discontinued, or reduced market shares, leading to higher supplier replaceability.

**Figure 5:**
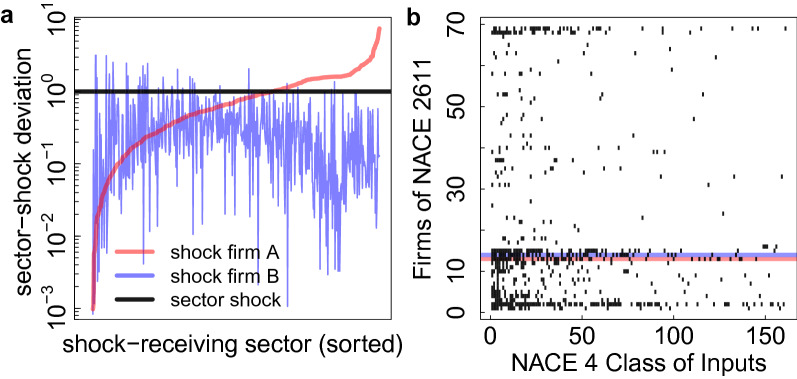
Importance of firm level analysis. (**a**) Effect on 568 NACE 4-digit industry sectors present in the data, following a general 18% shock of the entire NACE sector 2611 (Manufacture of electronic components). The x-axis denotes the 568 shock receiving sectors, the relative deviation of received shocks from the 18% homogeneous initial shock to sector 2611 is shown on the y-axis. The black line marks the reference scenario. The red line shows the relative deviation if firm A (in 2611) receives a 100% shock; the blue line shows the situation for a 59% shock to firm B. Both firm level shocks are equivalent in size to the 18% sector shock (reference). The particular choice of the defaulting companies lead to drastically different results on how other economic sectors are affected. (**b**) Most ubiquitous inputs (at NACE 4 level, columns) for 69 firms (rows) in NACE class 2611. Clearly, even though all companies belong to the same sector, their input sector vectors are drastically different. Inputs of firm A (red, 50 distinct inputs) and firm B (blue, 49 distinct inputs) differ substantially (Jaccard index 0.25). Note that 18 firms have no input sectors (empty rows).

### Importance of firm level shock propagation

We demonstrate that it is necessary to use firm level data for any reasonable assessment of shock propagation in production networks, by comparing it to a situation where shocks are studied on an aggregated level. In Fig. 5a we show three different economic shock scenarios that affect the NACE4 class 2611 (Manufacture of electronic components). All three initial shocks have the same size on the sector level, but affect different companies within the sector. The *initial shock* is exogenously applied and triggers a spreading of shocks in the production network. The *received shock* is the shock each sector receives as a result of the propagation of the initial shock. Initial shock size is measured as the fraction of the sector’s overall strength, ($s^\text {2611}=\sum _{i|p_i = 2611}s_i$), affected by the initial shock. The first shock scenario serves as a reference scenario. It applies a homogeneous initial shock of 18% (reduced production) to all of the 69 firms in sector 2611. The received shock of all 568 NACE 4 sectors present in the data (shown on the x-axis) marks the reference response. The received shocks are shown on the y-axis (in log-scale) as a fraction of this reference response, and by construction, the received shock for the homogeneous scenario is 1 (black line). In the second scenario a 100% shock (failure) is applied to a single firm, A, the received shocks in the different sectors (given as a fraction of the reference scenario) are shown as the red line. Sectors are sorted w.r.t. received shock sizes in this scenario (A). The third shock scenario applies a 59% shock to firm B, the responses are shown by the blue line. Firm A is a plateau firm, firm B is not. Clearly, the specific choice of the defaulting company within the sector has a drastic effect on how the different economic sectors are affected. Note that on the sector level the initial shocks are indistinguishable. In other words, *homogeneous* sector shocks (aggregated view) yield a grossly false picture of the true shock propagation and thus systemic risks. As so often in networked dynamical systems: details do matter. If studying shocks on the sector level was sufficient, the three scenarios should be strongly correlated. However, the figure suggests that the relative deviations from the sector shock (blue and red line) are negatively correlated with $-$19% ($p=5\cdot 10^{-6}$). In Supplementary Section S15 we show the actual received shocks (not their deviations from the reference scenario).

One obvious reason for these large differences between scenarios A and B is made clear in Fig. 5b, where we see for all companies in NACE sector 2611 the inputs at the NACE 4-digit level. Firms (rows) are sorted w.r.t. similarity of their inputs (columns). Even though all companies belong to the same fine grained industry sector (NACE 2611) the sectors of their inputs vary substantially. The input sectors of Firm A (red shading) and firm B (blue shading) have only a small overlap, indicated by a Jaccard index of 0.25. Depending on which of two companies fails, different input sectors are affected. The Jaccard index for the customer sectors is even smaller (0.13). For details on customer sectors, see Supplementary Section S15. Among the 51 firms (that in NACE 2611 have NACE 4 classified inputs) 56% have no pairwise input sector overlap (Jaccard index of zero) and 85% have no pairwise customer sector overlap. This means that when choosing two firms randomly, the probability of having no common input (customer) sector is 56% (85%). Consequently, shocks to different firms within a sector must lead to different economic sectors being affected.

## Discussion

Based on the reconstruction of all relevant buyer-supplier relations between companies in an entire country from VAT data, we demonstrate that the real economy can not be viewed as a collection of separate supply chains, but is a tightly connected directed network that has a strongly (weakly) connected component, containing 26% (94%) of all companies. The network allows us to develop and compute an index for the approximate systemic risk every individual company poses to the economy, ESRI. We demonstrate explicitly that systemic risk based on aggregated sector level data yields a severely distorted picture.

We find that the 32 top risky companies contribute to 45 % of the entire systemic risk, the top 100 companies contribute to 74 %. Only 165 companies have more than 1% risk (i.e. affect more than 1% of the economies output). The 32 top risky companies (0.035% of all 91,595 companies) show extremely high systemic risk of about 23%. They are connected by a network of highly critical supply relations (plateau network). Of those, only a fraction appears to be inherently risky (e.g., because of size or high market shares). The average systemic risk of a Hungarian company is $\overline{\mathrm{ESRI}_i} = 0.00018$ (mean). The median is $1.7 \cdot 10^{-6}$.

Approximately a third of the top risky companies are at the periphery of the plateau network. These are small and inherit systemic risk from the core plateau companies, because they are critical suppliers to the core. They would not show high ESRI values if they supplied to other – non-risky – companies. This means that several of these smaller companies can be made less risky simply by increasing the number of suppliers of the specific good sold to the inherently risky companies. By analyzing changes of systemic risk from 2016 to 2017, we find that ESRI is relatively stable for most companies. However, several smaller companies change their ESRI from marginal to extreme, and vice versa. The reasons for this might be the risk-inheritance of (non-inherently risky) companies that start (or stop) supplying to inherently risky ones, or changes in market shares that affect their replaceability. We showed that to a large extent firm size (strength) is a bad predictor of firms’ systemic risk, because the position in the supply network matters more than size. Similarly, this has been observed in financial systemic risk in earlier studies^30,31,47,52^.

The presented study has limitations. The proposed measure is an economically motivated, straightforward quantitative measure for the systemic relevance of companies in a given production network. It treats up- and downstream impacts independently of each other. This doesn’t cause distortions in tree-like supply chains when suppliers are not replaced, however, in networks with strongly connected components —like the present one— it does. We showed that firms are affected by up- and downstream shocks simultaneously. In practice these shocks can potentially interact. For example, the lack of a critical input (down-stream shock) can translate into an upstream shock for suppliers of complementary inputs. Such second-order effects are not considered by the algorithm and their magnitude needs to be estimated in future work with more subtle algorithms. Nonetheless, ours is the first attempt to calculate firm level ESR for an entire national economy and the resulting index can be seen as a good first-order estimate for the systemic risk in production networks. We assume the standard proportional rationing mechanism, i.e. that all customers and all suppliers are treated as being equally important. Other rationing mechanisms, for example, prioritizing large firms, could lead to modified ESRI estimates. For the sector level, the effects of different rationing mechanisms have been analyzed in detail^48^. Further, for the implementation of the algorithm we assumed a data-driven replaceability index for each firm that is based on its market share (fraction of output within its NACE 4 class). This implies that firms with high (low) market shares are difficult (easy) to replace, see Supplementary Section S5. Further improvements of the ESRI would be possible by modelling the substitutability of inputs and the replaceability of suppliers in more detail. However, this comes at the cost of more parameters to calibrate, larger data needs, and significantly higher computational effort.

For assigning production functions to companies, we used a simple matching based on NACE 2-digit categories. Although we think that this is a good first approximation, we emphasize that this can be a source of error. Given the current data availability, an exact and objective mapping of production functions to companies is not feasible. Large-scale firm level surveys providing information on how strongly firms are affected by the lack of specific inputs could be a useful first step clarifying this point. We assume a static production network, which in reality, is a temporal network. With this simplification, seasonality effects in production and manufacturing are missed. Also, in the present implementation we don’t consider competition between companies that would result in a dynamic restructuring of the network. Moreover, it is technically hard to interpret the time index *t* in terms of how long shocks really need to spread and converge. For simplicity, we assume uniform spreading, i.e. all periods *t* are of the same abstract length. Imports, exports, and production networks of other countries are not considered due to lack of data. This is a limitation since Hungary is a small, open economy with significant exposures to shocks in the global economy. In principle the vulnerability to initial shocks from import and export relationships can be considered in our framework by using coarse grained sector level import-export information. Despite these limitations, our findings have a number of policy implications.

### Monitoring

The economic systemic risk index for individual companies, ESRI, allows countries to use VAT data to identify critical companies in the economy and their critical supply relations (via their marginal ESRI contributions, see^32^). Our findings indicate that only a few firms pose a substantial risk to the overall economy. These should be monitored closely if they produce goods of societal relevance. In particular, sharp increases in firms’ systemic riskiness can be monitored over time. Several countries are currently implementing “supply chain due diligence” laws, for example, Germany^49^ or the USA^50^. The possibilities for systemic risk monitoring as presented in this paper should be kept in mind, when designing new regulation.

### Systemic risk mitigation

A straightforward way to increase resilience in the real economy is to introduce supply chain redundancies, where risk is reduced by altering the network structure and thereby reducing the probability for inherently systemically risky firms to suffer failures that result from a lack of inputs. We have shown that a simple network analysis allows us to identify the inherently risky firms. For those, contingency plans should be established to buy time for changing suppliers or to build alternative production capacities. The change of ESRI over time would allow countries to monitor if implemented policies really increase resilience levels of the economy.

### Avoiding risk concentration

In^32^ it has been shown how incentive schemes can be designed to make financial networks safer without making them less efficient. A similar scheme could be devised for production networks by de-incentivizing critical supply relations. A simple scheme would be to have at least one back-up supplier for every critical product that is shipped to inherently risky firms. Like in regulations of the financial sector, risk concentration can be avoided by demanding that no customer should have more than a certain supply exposure (e.g. 10% of total exposure).

### Inventory buffers

A natural possibility is to introduce mandatory inventory buffers for systemically risky companies, to ensure production in situations where a set of critical suppliers default.

### Make economic systemic risk visible for firms

Today, companies usually manage their direct suppliers. Most companies do not know their higher-order dependencies in the production network^51^. It is conceivable that it could be highly beneficial to many producing companies to obtain a better overview on their actual supply and customer risks. If countries provided a more global view on supply chains to identify critical situations, this could lead to deeper and more proactive systemic supply chain management, that would increase overall economic resilience by using market mechanisms.

Policies and regulatory measures of this kind might run contrary to the hunt for efficiency that dominated the last decades, since supplier relations are time- and cost intensive. More resilience does not pay off in the short term. It would be a fascinating question to see to what extent the economy could be made more resilient by not making it less efficient, i.e. to maximize the resilience-gain per link. For financial networks the potential of designing networks with lower systemic risk, but comparable economic function was recently highlighted in^52,53^. A related question is whether service-based economies are more resilient than production-based ones because services typically require less critical suppliers. Instead of asking whether large economies are more stable than small ones^43^, our methodology, given data from other countries, could contribute to the question, whether production-based economies or economies with high levels of input-redundancy are more resilient.

## Methods

We show in detail all equations necessary to calculate the ESRI for any firm in the production network, given the supply network *W*, the industry affiliation vector *p* and the defined sets of essential products $\mathscr {I}_i^\text {es}$ and non-essential products $\mathscr {I}_i^\text {ne}$ for each firm *i*. The derivation of the equations are shown in Supplementary Section S6. For the algorithm implementation we formulate the recursions Eqs. (2) and (3) directly in terms of the relative production levels $h^\text {d}(t)$ and $h^\text {u}(t)$. First, to rewrite the recursive update equations Eqs. (2) and (3) more compactly, we compute the downstream impact matrix $\Lambda ^\text {d}$ as

5 $$\begin{aligned} \Lambda _{ji}^\text {d} = {\left\{ \begin{array}{ll} \Lambda ^{d1}_{ji} \qquad \text {if} \; p_j \in \mathscr {I}_i^\text {es}, \\ \Lambda ^{d2}_{ji} \qquad \text {if} \; p_j \in \mathscr {I}_i^\text {ne}, \end{array}\right. } \end{aligned}$$

whereby elements of $\Lambda ^{d1}$ and $\Lambda ^{d2}$ are defined as

6 $$\begin{aligned} \Lambda ^{d1}_{ji} = {\left\{ \begin{array}{ll} \frac{W_{ji}}{\sum _{l=1}^{n} W_{l i} \delta _{p_{l},p_{j}} } \quad \text {if} \; W_{ji}> 0 , \\ \qquad 0 \quad \qquad \; \text {else}, \end{array}\right. } \qquad \text {and} \qquad \Lambda ^{d2}_{ji} = {\left\{ \begin{array}{ll} \frac{W_{ji}}{\sum ^{n}_{l=1}W_{li} } \qquad \quad \text {if} \; W_{ji} > 0 , \\ \quad 0 \qquad \qquad \;\; \: \text {else}. \end{array}\right. } \end{aligned}$$

The element, $\Lambda _{ji}^\text {d}$, determines the fraction of production firm *i* loses if firm *j* stops supplying to firm *i*. It is the relative exposure of firm *i*’s production to firm *j*. This impact varies depending on if *j* is supplying an essential good (Leontief impact) or a non-essential good (linear impact). The upstream impact matrix is computed as

7 $$\begin{aligned} \Lambda ^\text {u}_{ji} = {\left\{ \begin{array}{ll} \frac{W_{ij}}{ \sum _{l=1}^n W_{il} } \qquad \text {if} \quad W_{ij}>0 , \\ \quad 0 \qquad \quad \; \; \text {else}. \end{array}\right. } \end{aligned}$$

The element $\Lambda ^\text {u}_{ji}$ determines the fraction of production firm *i* loses if firm *j* stops buying from firm *i*. It is the exposure of firm *i*’s production to firm *j*. For the upstream propagation all impacts are linear.

Next, we initialize the recursion by specifying the initial shock to the network with the help of the exogenous shock parameter $\psi _i$ that determines for each firm *i* the fraction of production remaining when the initial shock occurs. The shock can be interpreted as any sort of operational or economic failure that leads to a (temporary) stop of production of firm *i* and consequentially a stop of purchases and sales. To calculate the ESRI$_{j'}$ of firm $j'$ we set $\psi _{j'}=0$ (0% production remaining) and $\psi _i=1$ (100% production remaining) for all other firms $j\ne j'$. Note that the homogeneous initial shock of 18% to all 69 firms in sector 2611, is implemented by setting $\psi _j=0.82 \; \forall \; j \,|\, p_j = 2611$.

The algorithm is initialized at $t=0$, defining $\psi$ as above and setting $h^\text {d}(0) = h^\text {u}(0) = 1$. Then, we iterate sequentially the recursive update equations Eqs. (8)–(12) while the production levels $h^{\text {d}}(t)$ and $h^{\text {u}}(t)$ are changing.

First, at each iteration *t* we calculate the current market share of each firm *j* in its industry $p_j = k$ as

8 $$\begin{aligned} \sigma _j(t) = \min \left[ \frac{s^\text {out}_j(0)}{\sum _{l=1}^{n} s^\text {out}_l(0)h_l^d(t) \delta _{p_l,p_j}},1 \right] \quad . \end{aligned}$$

The current market share $\sigma _j(t)$ is used as a proxy for how replaceable firm *j* is for its buyers. This implies that firms with low market share are to a certain extent replaceable, whereas firms with market shares higher than 50% are not replaceable in the short term. See Supplementary Information S5 for more details.

Second, we calculate for each firm *i* the fraction of essential and non-essential inputs that are still available at time *t*. For each essential input $k\in \mathscr {I}_i^\text {es}$ we update the fraction of available input, *k*, according to

9 $$\begin{aligned} \tilde{\Pi }_{ik}(t) = 1 - \sum _{j=1}^n \sigma _j(t) \Lambda ^{d}_{ji} \left( 1-h_j^\text {d}(t) \right) \, \delta _{p_j,k} \quad . \end{aligned}$$

Initially 100% of input *k* is available at $t=0$. From this we deduct for each supplier *j*, the lost fraction of production of supplier *j*, $1-h_j^\text {d}$ times the fraction of input *k* that is supplied by firm *j* to firm *i*, $\Lambda ^{d}_{ji}$, *j*, times the replaceability factor $\sigma _j(t)$. The remaining fraction of all non-essential inputs, $k\in \mathscr {I}_i^\text {ne}$, can be updated jointly as an auxiliary product category $k'$ according to

10 $$\begin{aligned} \tilde{\Pi }_{ik'}(t) = 1 - \sum _{k\in \mathscr {I}_i^\text {ne}} \sum _{j=1}^n \sigma _j(t) \Lambda ^{d}_{ji} \left( 1-h_j^\text {d}(t) \right) \, \delta _{p_j,k} \quad . \end{aligned}$$

The joint update of non-essential inputs as single category $k'$ is possible, because the GL production function does not treat non-essential inputs as complementary inputs. Note that $\tilde{\Pi }_{ik'}(t)$ can only drop to zero for a firm where all inputs are non-essential and the replaceability coefficient is 1 for all *j*, because then $\sum _{j\in \mathscr {I}^{ne}} \Lambda ^\text {d}_{ji} =1$.

Third, we update for each firm *i* its relative production level determined by the available fraction of inputs

11 $$\begin{aligned} h_i^{\text {d}}(t+1) = \min \left[ \min _{k \in \mathscr {I}_i^\text {es}} \Big (\tilde{\Pi }_{ik}(t) \Big ), \; \tilde{\Pi }_{ik'}(t) , \psi _i \right] \quad . \end{aligned}$$

This is the fraction of the original production (at $t=0$) firm *i* can still produce at iteration $t+1$, given the state of its suppliers.

Fourth, we update the relative production level of firm *i*, determined by the upstream shocks received from its buyers by

12 $$\begin{aligned} h^u_{i}(t+1) = \min \left[ \sum _{j=1}^n \Lambda ^\text {u}_{ji}h^\text {u}_j(t), \psi _i \right] \quad . \end{aligned}$$

This is the fraction of the original production (at $t=0$) firm *i* can still produce at iteration $t+1$, given the state of its buyers. Note that setting $\psi _{j'}=0$ at $t=0$ will affect the neighbouring nodes of $j'$ in the same way as setting $h^\text {d}_{j'}(1)=h^\text {u}_{j'}(1)=0$ in Eqs. (2) and (3). The update equations are iterated until the algorithm converges at time, *T*, where $\epsilon =10^{-2}$ is chosen as the convergence threshold. Once all changes in production level are smaller than $\epsilon$ the propagation stops and the corresponding time point of convergence is *T*. As mentioned in the introduction, Eqs. (11) and (12) are iterated independently of each other. We set the final amount of production for each firm *i* to $h_i(T)=\min (h_i^d(T),h^u_i(T))$.

The economic systemic risk index, ESRI$_{j'}$, of firm $j'$ is now calculated as the weighted sum of lost output in the production network in response to the (temporary) failure of firm $j'$

13 $$\begin{aligned} \mathrm{ESRI}_{j'} = \sum _{j=1}^{n} \frac{s^\text {out}_j }{\sum _{l=1}^n s^\text {out}_l }\left( 1-h_j(T) \right) \quad . \end{aligned}$$

Note that the interpretation is conditional on the assumption that the supply of inputs is only replaced in size of the replaceability factor $\sigma$ and the demand of the failing firm is not replaced at all. Therefore, this is a short term perspective that neglects the restructuring of the network over time. The ESRI should be interpreted rather as an economically motivated metric to rank firms with respect to their systemic importance and not as a precise forecast of the actually lost total output.

Note that the Hungarian production network does not compromise all sales and purchases of firms based in Hungary, due to the reporting threshold and international transactions. When calculating the up- and downstream impact matrices, $\Lambda ^\text {d}$ and $\Lambda ^\text {u}$, we over-estimate the impacts single links have. The upstream impact from *j* to *i*, $\Lambda _{ji}^\text {u}$, associated to link $W_{ij}$ needs to be adjusted for the sales transactions that are not included in the observed network, *W*. These residual sales transactions are included in the revenue of firm *i*. Thus, for the empirical application of the algorithm we re-weight the element $\Lambda ^\text {u}_{ij}$ by the factor $s_j^\text {out} / r_j(0)$, where the numerator is the output recorded in the observed production network and $r_j(0)$ is the revenue of firm *j* for the respective year. Similarly, we re-weight $\Lambda _{ji}^\text {d}$ by $s_j^\text {in} / c_j(0)$, where the numerator is the volume of purchased inputs within the observed production network and $c_j(0)$ denotes the material costs in the respective year. We use income statement data of the years 2017 and 2016 of the firms, wherever available to make this adjustment. For those firms where this is not available we apply a factor of 1. We report that $(\sum _{i=1}^{n}s_i^\text {out}) / (\sum _{i=1}^{n}r_i(0))=0.60$ and $(\sum _{i=1}^{n}s_i^\text {in} )/ (\sum _{i=1}^{n}c_i(0))=0.76$. Omitting this re-weighting would lead to a significant overestimation of ESRI. A potential error arises by re-weighting for firm *i* $\Lambda _{ji}^\text {d}$ by the same factor for all input suppliers *j*, since we do not know which input types the unobserved quantity $c_j(0)-s_j^\text {in}$ compromises.

## Supplementary Information

Supplementary Information.
