# Supplementary material for: Quantifying firm-level economic systemic risk from nation-wide supply networks
Source: Sci Rep. 2022 May 11;12:7719. doi: 10.1038/s41598-022-11522-z (PMC9092945; doi:10.1038/s41598-022-11522-z)
Supplement: Supplementary file 1 — Supplementary Information. [file 41598_2022_11522_MOESM1_ESM.pdf]

# Supplementary Information
# Quantifying firm-level economic systemic risk from nation-wide supply networks

Christian Diem[1,2], András Borsos[1,3,4], Tobias Reisch[5,1], János Kertész[4,1], Stefan Thurner[5,1,6*]

[1]Complexity Science Hub Vienna, Josefstädter Strasse 39, A-1080 Vienna, Austria
[2]Institute for Finance, Banking and Insurance, Vienna University of Economics and Business, Welthandelsplatz 1, A-1020 Vienna, Austria
[3]Financial Systems Analysis, Central Bank of Hungary, Szabadság tér 9, 1054 Budapest, Hungary
[4]Department of Network and Data Science, Central European University, Quellenstrasse 51, A-1100 Vienna, Austria
[5]Section for Science of Complex Systems, CeMSIIS, Medical University of Vienna, Spitalgasse 23, A-1090, Austria
[6]Santa Fe Institute, 1399 Hyde Park Road, Santa Fe, NM 85701, USA
[*]stefan.thurner@muv.ac.at

## S1 Relation to I-O analysis

On the industry level studies on the propagation of shocks in production networks exist at least since the famous Leontief Input-Output analysis[1–3]. In[4] it is shown theoretically that small shocks to individual sectors can have effects on aggregate output. Refs.[5–7] confirm this finding.[8] assess higher order shocks from the Hurricane Katrina disaster. More recently,[9] investigated how demand and supply constraints on the sector level affect GDP. In[10] it is modeled how shocks spread through the Tanzanian supply and transport network. The sector level analysis produced valuable insights, but it has certain limitations due to the nature of the underlying data. Only recently high-quality large-scale firm level data has become available that allows for new insights.

The methodology presented in this paper addresses three usual shortcomings of sector level analyses. First, the data shows that even within fine grained industry classifications (NACE 4) firms tend to have considerably heterogeneous input sectors and customer sectors. In fact when comparing the pairwise input (customer) sectors of firms we see that for all firms in NACE 2611 (Manufacture of electronic components) 56% (85%) have no overlap of input (customer) sectors even though they belong to the same NACE 4 category, see Fig. 5 in the main text and Supplementary Section S15. Second, this intra-sector heterogeneity can lead to inaccurate results when using them for assessing shock propagation in production networks. Especially if the initial crises scenario does not affect all firms within a sector to the same extent (for example in the current COVID-19 crises). Our proposed framework takes this into account and yields different cascades for shocks which would appear to be the same at industry level, but are actually distributed heterogeneously among firms within an industry. Fig. 5 a) shows in a simple example how two cascades that are the same on the industry level lead to very different impacts on other firms and industry sectors. Third, in contrast to sector level models, each firm in our approach has a specific production function based on its industry classification that is calibrated to the observed individual input vector of the respective firm; see Supplementary Section S4 for more information on the calibration of production functions. This matters especially since we show in Fig. 5 b) that firm input vectors, even in the fine grained industry classifications, vary on the sector level. The same is shown for customer sectors, see Supplementary Section S15, Fig. S9 b).

Since, data has become available also firm level analysis has been performed. Ref.[11] uses a generative model for firm level production networks that matches degree distributions better than scale-free frameworks. Belgian VAT data has been used to study productivity shocks to individual firms and their effects on aggregate output with a computeable equilibrium model[12]. The theoretical study[13] shows that shocks propagate widely as soon as production function have a "Leontief" component, but not when they are of pure Cobb-Douglas type. Probably the closest study to ours,[14], investigates how firm level shock propagation in response to an initial shock –the great earthquake in Japan in 2011– based on an estimate of the Japanese production network. However, they focus on effects on aggregate output and do not compute firm level systemic risk.

## S2 Relations to financial systemic risk

In the area of financial networks systemic risk has been extensively studied for about two decades[15,16]. The importance of being able to measure systemic risk of single firms (banks, insurance, funds) became apparent in the 2008 financial crises and the European government debt crises 2012. Initial systemic risk assessment methodologies have been shown by, for example,[17–19] they have been refined to macroprudential stresstesting models that can assess the effects of adverse macro-economic scenarios[20] and to measure the impact single banks have on the entire network[21,22]. A wide range of studies revealed various properties of financial networks that improve the understanding of how systemic risk emerges, for example, the role of network topology[23,24], the role of diversification of external assets[25], or the role played by large banks[26]. These aspects are crucial for the management of systemic risk. The design of regulatory policies to address the adverse economic and societal effects of systemic risk has strongly benefited from these academic contributions. However, there are important differences between financial and production networks and how stress is spreading there, that require adaptions to how systemic risk and the spreading of shocks is defined and modelled. For example, in production networks in- and out-links affect heterogeneous production process in contrast to stock quantities like equity or liquidity buffers in financial networks. Further, production networks tend to be orders of magnitude larger than banking networks. Here we extent the ideas of measuring systemic risk in financial networks[21,22,27] to a more general framework of companies in a production network at a national scale. To estimate it we use micro level VAT data of Hungary[28].

## S3 Industry classification schemes

Around the world there are millions of different products produced, all having small distinctive features. However, to allow for a meaningful statistical and economic analysis these products are usually grouped and categorized into product classes according to some common features. Examples are the Cooperative Patent Classification (CPC) and the EU's classification of products by activity (CPA). They contain 2647 and 3142 classes, respectively. The second kind of classification aims at grouping companies that produce these product groups based on common activities. Industry classifications have various levels of granularity, for example the International Standard Classification of All Economic Activities, ISIC, has 88 categories at the 2-digit and 419 at the 4-digit level; the Statistical Classification of Economic Activities in the European Community NACE has 88 categories at the 2-digit and 615 4 digit level; the North American Industry Classification System, NAIC, features 1057 categories at the 6-digit level. Note that the CPA and NACE classifications can be mapped onto each other. A concise overview can be found in[29].

In practice however, these classifications are not linked to the single supply transactions, $W_{ij}$. As outlined in the introduction of the main-text economists typically resort to the assumption that each company $i$ produces one of $m$ possible different products, identified by the firm's industry classification. In mathematical notation we use the industry affiliation vector, $p$, to identify the industry affiliation $p_i \in \{1,2,\dots,m\}$ for each firm $i$ in its entries. On the modelling side, this simplifying assumptions amounts to reducing a firm level input-output matrix, with each column determining the necessary inputs for each product firm $i$ produces, to a single input vector. In mathematical terms this simplification reduces the output vector $x_{i1}, x_{i2}, \dots, x_{im}$ of company $i$ to a scalar output $x_i$ of type $p_i$. Consequentially, the input matrix ($\Pi^i$ —with element $\Pi^i_{kl}$ determining the amount of input, $k$, used to produce output $x_{il}$— reduces to an input vector, $(\Pi_{i1}, \Pi_{i2}, \dots \Pi_{im})$ and the production function which in reality is a map $f : \mathbb{R}^m_+ \to \mathbb{R}^m_+$ reduces to $f : \mathbb{R}^m_+ \to \mathbb{R}_+$. In short, a multi-layer network is aggregated to a single network layer, where each buyer-supplier relation, $W^1_{ij}(t), W^2_{ij}(t), \dots W^m_{ij}(t)$, is simplified to a single transaction, $W_{ij}(t)$, of product, $p_i$.

The reduction of a distinct input and customer vector for each product a firm produces to a single vector for each firm leads to a blur of the up- and downstream contagion. This is illustrated with the case where firm $i$ produces two different products, $A_i$ and $B_i$, requiring each two types of inputs. $A_i$ requires $\alpha$ and $\gamma$, $B_i$ requires $\beta$ and $\gamma$. If either the suppliers of input $\alpha$ or input $\beta$ fail to deliver only the production of one of the two products $A_i$ or $B_i$ is adversely affected. Consequently, a customer that only buys $A_i$ is affected only in case input $\alpha$ is becoming scant, while another customer who only buys $B_i$ is affected in the case where input $\beta$ is not available. In the aggregated picture where there is only one abstract product of type $p_i$, both customers are affected by a loss of input $\alpha$ or $\beta$. Note in the case of Leontief production functions, if either input $\alpha$ or $\beta$ is not available, this would affect the entire production of firm $i$, even though in reality it would only affect the production of either $A_i$ or $B_i$. Similarly, for upstream contagion, if only one of the two customers stops buying, just the suppliers of either $\alpha$ or $\beta$ are affected but not both. The presented framework can be easily extended to account for this as soon as appropriate data becomes available.

## S4 Production functions

The mere existence of a buyer-supplier connection does not say much about how their production processes depend on each other, i.e. how the failure of one affects the other. This exposure depends primarily on the type of goods and services delivered and how these specific intermediate products (inputs) are used in combination with each other. Naturally, some inputs are more critical for specific production processes than others. We need to know the type of goods and services that are delivered

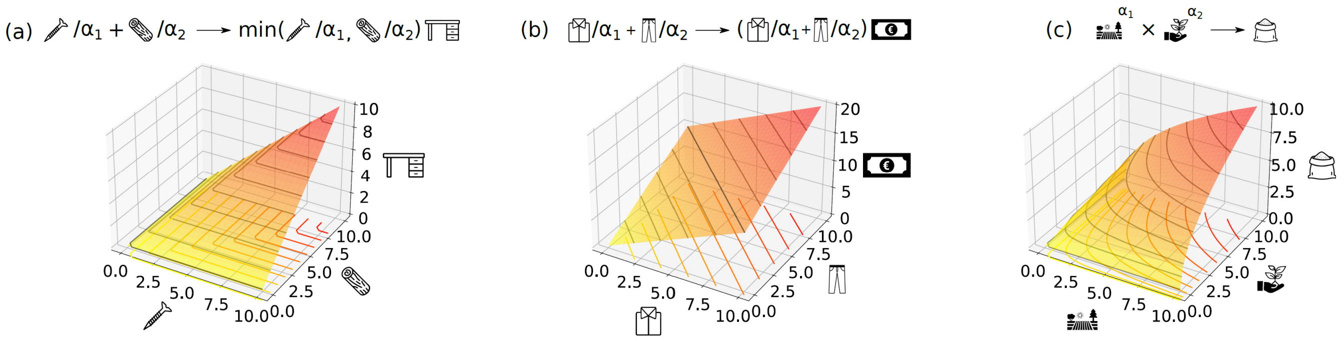


**Figure S1.** Illustration of production functions. a) The Leontief production function, $x_i = \min\left(\frac{\Pi_{i1}}{\alpha_{i1}}, \frac{\Pi_{i2}}{\alpha_{i2}}\right)$, is constrained by the least available input. We illustrate this with a carpenter building tables. She requires an exact amount of wood and nails for every table. b) Linear production function of two inputs, $x_i = \frac{\Pi_{i1}}{\alpha_{i1}} + \frac{\Pi_{i2}}{\alpha_{i2}}$. For a linear production function the output is proportional to a linear combination of the inputs, here illustrated by a retail company, selling shirts and trousers. If shirts are unavailable as an input, the revenue generated by trousers is not affected. c) Cobb-Douglas production function, $x_i = \beta_i \Pi_{i1}^{\alpha_{i1}} \cdot \Pi_{i2}^{\alpha_{i2}}$, with $\alpha_1 + \alpha_2 = 1$. The Cobb-Douglas production function can be interpreted as an intermediate case, allowing for *partial substitution*. Exemplified by the production of crops, the amount of land used for the crops can be reduced if e.g. more fertilizer is invested. However, with the complete lack of either production factors, any production is impossible.

and an approximations for the firms' production processes to model these exposures. As outlined in the main text, production processes of firms in the economics literature are commonly modelled with production functions. The standard choices are the constant elasticity of substitution production function (CES)[30] and its special cases, the Cobb-Douglas and the Leontief production function. Note the linear production function is also a special case of CES.

**Intuition.** In Fig. S1 we show a schematic visualisation of the three special cases. Figure S1 a) shows a Leontief production function for firm $i$, with two types of input, $\Pi_{i1}$ (screws) on the x-axis, $\Pi_{i2}$ (wood) on the y-axis, and the resulting production level, $x_i$ (tables) on the z-axis,

$$x_i = \min\left(\frac{1}{\alpha_{i1}}\Pi_{i1}, \frac{1}{\alpha_{i2}}\Pi_{i2}\right) \quad .$$

The amount of tables produced depends on the availability of both products according to a fixed production recipe, $\alpha_{i1}$ screws and $\alpha_{i2}$ cubic meters of wood. Since, we only know the volumes (= price $\times$ quantity), the interpretation changes such that $\alpha_{i1}$ is the money spent on screws as a fraction of the table's value and $\alpha_{i2}$ is the money spent on wood as a fraction of the table's value. In this case, if 50% less of one of the inputs is available, only 50% of the tables are produced. In industrial processes the production recipes encoded in the $\alpha_{ik}$ are commonly used for production planning and management.

Figure S1 b) shows a linear production function of firm $i$ with two different types of inputs, $\Pi_{i1}$ (trousers) on the x-axis and $\Pi_{i2}$ (jackets) on the y-axis and the resulting production level, $x_i$ (sales of trousers and jackets) on the z-axis,

$$x_i = \frac{1}{\alpha_{i1}}\Pi_{i1} + \frac{1}{\alpha_{i2}}\Pi_{i2} \quad .$$

It is clear that "production" in this case is possible with only one of the inputs. The coefficients $\alpha_{i1}$ and $\alpha_{i2}$ determine how important each of the inputs is for the output. The ratio, $\alpha_{i1}/\alpha_{i2}$, determines how much of input 1 needs to be added to substitute the one unit loss of input 2 to keep the same level of production. In our example the inputs are equally important. The linear production function can be used to reasonably describe a trades business or services. In the case of a trade business the coefficients $\alpha_{ik}$ correspond to the *markup* firm $i$ charges when re-selling input $k$ to customers. Similarly, for the delivery of services, inputs are not used jointly as intermediate inputs to produce a new physical product, but to support the creation process. Illustrative examples are a hairdresser, running out of shampoo can still cut hair, a consulting firm running out of office supplies that still can produce powerpoint slides, a software developing company facing delays of new computers that can still produce new code on the old machines. Even though the processes are not becoming impossible to execute, they become less efficient or reduce in quality, thus leading to effectively less output. Text books usually state that linear production functions are of limited or no practical use[31]. However, it is obviously a sensible approximation for the above mentioned situations. Clearly, it fails to describe the production process of physical goods where inputs are inherently combined and transformed to become an output of a new type.

Figure S1 c) shows a Cobb-Douglas production function for firm $i$ with two different types of input, $\Pi_{i1}$ on the x-axis, $\Pi_{i2}$

on the y-axis, and the resulting production level, $x_i$ on the z-axis,

$$x_i = \beta_i \Pi_{i1}^{\alpha_{i1}} \cdot \Pi_{i2}^{\alpha_{i2}} \quad .$$

It is clear that "production" is more efficient if both inputs are available, because the fewer of input one is available, the more of input two is needed to compensate for this loss and keeping the production level constant. In the extreme, an infinite amount of input one would be needed to completely substitute two. This is of course not possible in reality, but indicates that production with one input alone is not possible. In its original formulation it was not intended to describe production based on different intermediate inputs, but on labour ($l_i$) and capital ($c_i$), $x_i = b \cdot l_i^{\alpha_i} \cdot c_i^{1-\alpha_i}$[32]. Note that if the log is taken on both sides of the equation, this yields a linear production function. The interpretation is that if $\Pi_{i1}$ grows (decreases) by 1%, output will grow (decrease) by $\alpha_{i1}$ %, at the given production level. On the sector level $\alpha_{ik}$ can be set to the technical coefficients, derived from input-output tables[3,33], i.e., $\alpha_{ik} = \Pi_{ik}/x_i$. This is equally possible on the firm level.

**Interpretation in the context of shock spreading.** Usually the production function specifies how much goods and services (output), $x_i$, of type $p_i \in \{1, 2, \ldots, m\}$ firm $i$ can produce with a given amount of $m$ different intermediate products (inputs) $(\Pi_{i1}, \Pi_{i2}, \ldots \Pi_{im}) \in \mathbb{R}_+^m$, manufacturing equipment (capital) $c_i$, and its employees (labour), $l_i$. The production functions are usually used in a long-term perspective, where different inputs (traditionally labour and capital) can be substituted, by for example, using more machines and less labour. In practical terms this implies a change of a firm's production process. Such changes are usually not possible in the short-term and often need at least a few months (lead times for buying new machinery, designing the new business processes, hiring differently qualified employees, train employees on new machines or software, etc.). This is especially true for complicated production processes of complex goods. Thus, for short-term spreading of shocks caused by, for example, the failure of a supplier, we assume that the production processes are constant and we neglect capital and labour within the production function. Similarly, we assume that intermediate inputs can not be substituted in the short-term by simply buying more of a different input. In the long-term, however, certainly, missing inputs can be substituted by other inputs when the production process is sufficiently adapted. Consequently, for the analysis of shock propagation in the production network, we use the production functions to determine how much a firm can still produce in the short-term if a certain input is not available. As discussed in Supplementary Section S5, the assumption of replacing a failing supplier by another that can deliver a comparable input, has a strong impact of how shocks are spreading.

**Leontief-, linear-, and generalized Leontief production functions.** We now show the exact specifications of the production functions used to model the spreading of up- and downstream shocks in the economic systemic risk index, ESRI. Note that the calibrations are made for an observed production network, $W$, and an industry affiliation vector, $p$. These two objects determine the output volume of every firm and the volume of respective inputs purchased in the observed production network. We specifically use the linear production function for trades businesses and services companies, and a modified Leontief production function for firms with a physical production processes, to allow for a different treatment of inputs from *physical* production processes and from services.

We start from observed outputs and inputs of firms determined by the supply network, $W$, where $W_{ij}$ denotes the volume (value) supplied from firm $i$ to firm $j$ in the respective observation period. The input, $k$, of firm $i$ is mapped to its suppliers by,

$$\Pi_{jk}(t) = \sum_{i=1}^{n} W_{ij}(t) \delta_{p_i,k} \quad , \tag{1}$$

where $\delta_{a,b}$ is the Kronecker delta that is equal to 1, if $a = b$ and 0, otherwise. The output, $x_i = \sum_{l=1} W_{il}$, is the sum over all supply transactions to other firms, $l$.

First, we consider the Leontief production function for company $i$ as

$$x_i \quad = \quad \min\left[\frac{1}{\alpha_{i1}} \sum_{j=1}^{n} W_{ji} \delta_{1,p_j}, \frac{1}{\alpha_{i2}} \sum_{j=1}^{n} W_{ji} \delta_{2,p_j}, \ldots, \frac{1}{\alpha_{im}} \sum_{j=1}^{n} W_{ji} \delta_{m,p_j}\right] \quad . \tag{2}$$

Note that this formulation implies that all suppliers $j$ of $i$ supplying the same input, $p_j = k$ (industry classification) are treated as *perfect substitutes*. The corresponding parameters $\alpha_k$ for company $i$ are the *technical coefficients*,

$$\alpha_{ik} = \frac{\sum_{j=1}^{n} W_{ji} \delta_{p_j,k}}{\sum_{l=1}^{n} W_{il}} \quad . \tag{3}$$

$\alpha_{ik}$ determines the fraction of output that firm $i$ needs to spend on input $k$ to produce this respective output.

Second, we consider the simplified linear production function

$$x_i = \frac{1}{\alpha_i} \sum_{j=1}^{n} W_{ji} \quad , \tag{4}$$

with

$$\alpha_i = \frac{\sum_{j=1}^{n} W_{ji}}{\sum_{l=1}^{n} W_{il}} \quad . \tag{5}$$

It is clear that Eq. (4) is a special case of Eq. (2) when all product types are the same. For shock propagation this implies that the loss of an input affects the output proportional to the value of the inputs. Note that the linear production function has a natural interpretation for a trades business, where $1/\alpha_i - 1$ corresponds to the definition of the *markup* ($Markup = (Sale\ Price - Cost)/Cost$). In our 2017 dataset, trades businesses (NACE classes G45-G47) make up for 19.84% of firms. In practice distributors apply a different markup for different product groups. If sufficient data were available this can be simply corrected by applying a different markup for each product group. The general linear production function is

$$x_i = \frac{1}{\alpha_{i1}} \sum_{j=1}^{n} W_{ji} \delta_{1,p_j} + \frac{1}{\alpha_{i2}} \sum_{j=1}^{n} W_{ji} \delta_{2,p_j} + \cdots + \frac{1}{\alpha_{im}} \sum_{j=1}^{n} W_{ji} \delta_{m,p_j}) \quad . \tag{6}$$

Then, for trades businesses the parameters $\alpha_{ik}$ would be chosen such that $1/\alpha_{ik} - 1$ corresponds to the markup of product group $k$ for company $i$.

Third, we consider a generalized Leontief production function (GL) where only essential inputs are treated in the Leontief sense and non-essential inputs are treated in the linear sense. We denote the set, $\mathscr{I}_i^{\text{es}}$, that contains all essential input types $k$, entering production in a Leontief way, and the set $\mathscr{I}_i^{\text{ne}}$ that contains all non-essential input types $k$ entering in a linear way. Then, we define the modified Leontief production function as

$$x_i \quad = \quad \min \left[ \min_{k \in \mathscr{I}_i^{\text{es}}} \left( \frac{1}{\alpha_{ik}} \sum_{j=1}^{n} W_{ji} \delta_{p_j,k} \right), \beta_i + \frac{1}{\alpha_i} \sum_{k \in \mathscr{I}_i^{\text{ne}}} \sum_{j=1}^{n} W_{ji} \delta_{p_j,k} \right] \tag{7}$$

The parameter $\beta_i$ is defined as the production level that is attainable with only essential inputs $k \in \mathscr{I}_i^{\text{es}}$, i.e.,

$$\beta_i = \left( \sum_{l=1}^{n} W_{il} \right) \frac{\sum_{k \in \mathscr{I}_i^{\text{es}}} \sum_{j=1}^{n} W_{ji} \delta_{p_j,k}}{\sum_{j=1}^{n} W_{ji}} \quad . \tag{8}$$

As for the linear production function, the parameter $\alpha_i$ is the fraction of output spent on the value of inputs. It ensures that a lack of non-essential inputs interpolates between the full production level $x_i$ and $\beta_i$. It is clear that both the Leontief and the linear production function are special cases when either all inputs are essential or non-essential. Note that here non-essential means that production is not stopped when the input is lacking, but there is still an impact on output, i.e. the input is non-essential yet relevant. This formulation can be easily extended to cover the additional case that inputs are non-essential and non-relevant. In that case a third group of inputs needs to be specified and those inputs simply do not affect the production function. We leave this for future research.

## S5 Replaceability of suppliers

On shorter time horizons the mode of production can only be changed in relatively subtle ways, e.g., by using a given input from a different supplier and even that is sometimes not possible if goods are not highly standardized. However, major modifications to the production process itself are much harder, especially for sophisticated products. Detailed modelling of how different companies can be replaced by others (as suppliers) would require the detailed knowledge of inventory levels, production capacities of suppliers, and detailed product information (e.g. if two suppliers in the same industry can produce the same good). With such additional data available, a consistent modelling of supplier replacement could be implemented with a dynamic rewiring of the network, i.e., companies looking for new suppliers with idle production capacities or inventory to replace a failed supplier. However, it is difficult to model this behaviour in a realistic way, without many additional parameters. Such extended modelling efforts would be computationally significantly more expensive.

Only a few strategies in the firm level supply chain contagion literature tackle this challenge, however, generally all attempts suffer from limited data availability.[14] consider replaceability only between existing suppliers.[34] uses a different strategy and creates a measure of replaceability based on the weight of a given supplier in its costumers' production-related

cost. This measure assumes that if a supplier is an important part of a firm's production, then it is harder to find a substitute for it. Another, frequently used solution, is to distinguish between standardized goods (goods with a clear reference price listed in trade publications) and differentiated goods (goods with multidimensional characteristics) based on[35]. Although this distinction can be used as a categorical variable in econometric estimations, e.g.,[36] it is not informative regarding the extent of replaceability even for standardized products. Ref.[37] uses two other proxies to measure the specificity of suppliers: the level of R&D expenditures and the number of patents held by a firm. Unfortunately, these pieces of information are only relevant for a tiny fraction of companies and not at all applicable to the entire network of Hungarian firms.

We propose a different strategy that employs a straightforward, data driven way to construct a short-term supplier replaceability index based on intra-industry market shares. The basic intuition is that a supplier, having a small market share within its industry, on average should be relatively easy to replace by a small increase of the production of its competitors to cover the additional demand for their products. However, a supplier producing a considerable share of the goods in a given industry is more difficult to replace, as it is unlikely that its competitors can increase their production immediately. Having a large market share within an industry category in a country does of course not necessarily mean that a firm is large. It also needs to be taken into account that potential alternative suppliers in the given industry might also have experienced shocks during the contagion processes in the model. To make this approach more realistic, we take the deterioration in their production capacity into account. Here, it is important to distinguish between the two different sources that could cause reductions in the production level of these potential alternative suppliers. On the one hand, one should account for downstream shocks, i.e. shocks coming from the suppliers, which is a truly limiting disruption in their production. On the other hand, one should *disregard* the upstream shocks experienced by them, because demand shocks coming from customers are not actual restraints on their production (at least from the point of view of replacing reduced output of their competitors). We can also account for the fact that in the case of a system-wide crisis it might not be possible to find alternative suppliers; see Eq. (20) for the mathematical formulation of the supplier replaceability.

The interpretation of the replaceability of suppliers in our approach can be illustrated by the following example. Assume that a supplier with a 10% market share within its industry (after considering also its competitors' states) is responsible for 50% of the input required by one of its customers in the given input category. If the production level of this supplier drops to 80%, then the production of the customer will decrease by approximately 1% $(= 10\% \times 50\% \times (1-80\%))$. If we disregarded the possibility of replacing this supplier, the corresponding decrease in the firm's output would be 10%. This way, we are able to replace missing supplies w.r.t. the market conditions; i.e. our replaceability factor reflects not only the fact that the given input in this example can be bought from the remaining 90% of the market, but also acknowledge that this replacement might not be possible entirely, or it entails some costs. In future research the interaction of upstream and downstream shocks needs to be taken into account also for modelling the replaceability. This is important because, if some producers receive upstream shocks, they would have an additional capacity to supply other firms with this, which themselves might be suffering a downstream shock. If these idle supply and idle demand is matched, this would result in less contagion.

## S6 Details on the derivation of the recursion

We explicitly determine the relations for Eqs. (2-3) in the main text for the generalized Leontief, the Leontief, and the linear production functions. We use the term update equation and recursion interchangeably.

**Downstream recursion.** We derive the downstream update equation for the generalized Leontief production function from Eq. (7)

$$x_i^{\mathrm{d}}(t+1) = \min\left[\min_{k\in\mathscr{I}_i^{\mathrm{es}}}\left(\frac{1}{\alpha_{ik}}\sum_{j=1}^{n}W_{ji}h_j^{\mathrm{d}}(t)\delta_{p_j,k}\right), \beta_i+\frac{1}{\alpha_i}\sum_{k\in\mathscr{I}_i^{\mathrm{ne}}}\sum_{j=1}^{n}W_{ji}h_j^{\mathrm{d}}(t)\delta_{p_j,k}\right] . \tag{9}$$

It is convenient to work with a direct recursion with $h_i^{\mathrm{d}}(t+1)$ on the left hand side and $h_j^{\mathrm{d}}(t)$ on the right hand side. Thus, we divide Eq. (9) on both sides by $x_i(0) = \sum_{l=1}^{n} W_{il}$,

$$h_i^{\mathrm{d}}(t+1) = \min\left[\min_{k\in\mathscr{I}_i^{\mathrm{es}}}\left(\frac{1}{x_i(0)}\frac{1}{\alpha_{ik}}\sum_{j=1}^{n}W_{ji}h_j^{\mathrm{d}}(t)\delta_{p_j,k}\right), \frac{1}{x_i(0)}\left(\beta_i+\frac{1}{\alpha_i}\sum_{k\in\mathscr{I}_i^{\mathrm{ne}}}\sum_{j=1}^{n}W_{ji}h_j^{\mathrm{d}}(t)\delta_{p_j,k}\right)\right] . \tag{10}$$

After a few simplifications (see Eq. (24-26)) we can write more compactly

$$h_i^{\mathrm{d}}(t+1) = \min\left[\min_{k\in\mathscr{I}_i^{\mathrm{es}}}\left(\sum_{j=1}^{n}\Lambda_{ji}^{d1}h_j^{\mathrm{d}}(t)\delta_{p_j,k}\right), \tilde{\beta}_i+\sum_{k\in\mathscr{I}_i^{\mathrm{ne}}}\sum_{j=1}^{n}\Lambda_{ji}^{d2}h_j^{\mathrm{d}}(t)\delta_{p_j,k}\right] . \tag{11}$$

The elements of the matrix $\Lambda^{d1}$ are defined as

$$\Lambda_{ji}^{d1} = \begin{cases} \frac{W_{ji}}{\sum_{\iota=1} W_{\iota i}\delta_{p_\iota,p_j}} & \text{if } W_{ij} > 0 \quad , \\ 0 & \text{else} \end{cases} \quad , \tag{12}$$

the elements of $\Lambda^{d2}$ are

$$\Lambda_{ji}^{d2} = \begin{cases} \frac{W_{ji}}{\sum_{l=1} W_{li}} & \text{if } W_{ij} > 0 \quad , \\ 0 & \text{else} \end{cases} \quad . \tag{13}$$

and $\tilde{\beta}_i$ is simply the relative fraction of production possible with only essential inputs $k \in \mathscr{I}_i^{\text{es}}$. Note that the Leontief production function (Eq. 2) and the linear production function (Eq. 4) are special cases, where either only the first, or the second part of Eq. (11) is present.

The elements of the Leontief downstream impact matrix, $\Lambda_{ji}^{d1}$, capture the impact that the failure of firm $j$ has on firm $i$, given that $j$ is supplying a "Leontief input" (essential) to firm $i$. Note that $\Lambda_{ji}^{d1} = 1$ means that firm $j$ is the only supplier of goods of type $p_j$ to firm $i$. Similarly, the elements of the linear downstream impact matrix, $\Lambda_{ji}^{d2}$, capture the impact the failure of firm $j$ has on firm $i$ if $j$ is supplying a "linear input" (non-essential) to firm $i$. Note that the case $\Lambda_{ji}^{d2} = 1$ is only possible if firm $j$ is the only supplier of firm $i$. This difference gives already a glimpse on the fact that the Leontief production function leads to much higher contagion levels than the linear one. Further, the case of $\Lambda_{ji}^{d1} = 1$ becomes more frequent if the product types (industry classifications) become more fine grained. This leads to the intuitive behaviour that companies producing scarce and essential (to many firms) resources, are expected to have a high systemic risk index.

As will become visible in Supplementary Section , for the ease of numerical implementation, we split the updating of Eq. (11) into two parts. First, we define the unified downstream impact matrix

$$\Lambda_{ij}^{\text{d}} = \begin{cases} \Lambda_{ji}^{d1} & \text{if } p_j \in \mathscr{I}_i^{\text{es}} \\ \Lambda_{ji}^{d2} & \text{if } p_j \in \mathscr{I}_i^{\text{ne}} \end{cases} \tag{14}$$

where $k \in \mathscr{I}_i^{\text{es}}$ contains all inputs $k$ that are essential to firm $i$ and $k \in \mathscr{I}_i^{\text{ne}}$ contains all inputs $k$ that are non-essential to firm $i$. Again, firms having a pure Leontief or linear production function are special cases. Second, we define the relative share of input $k$, available to firm $i$ at time $t$ as

$$\tilde{\Pi}_{ik}(t) = \sum_{j=1}^{n} \Lambda_{ji}^{d} h_j^{\text{d}}(t)\delta_{p_j,k} \quad .$$

Consequently, the $n \times m$ matrix, $\tilde{\Pi}(t)$, contains in row $i$ the relative input vector at $t$ for firm $i$. Relative refers to the initial state $t = 0$. Hence, $\tilde{\Pi}(t)$ can be updated by a matrix multiplication, $(\Lambda^{\text{d}})^\top P$, where $P \in \{0,1\}^{n\times m}$ is defined as

$$P_{ik} = \begin{cases} 1 & \text{if } \; p_i = k \quad , \\ 0 & \text{otherwise} \quad . \end{cases}$$

Note that —due to the definition of $\Lambda^{\text{d}}$— for the inputs $k \in \mathscr{I}_i^{\text{es}}$ the relative input, $\tilde{\Pi}_{ik}(t)$, is equal to 1 if no supplier defaulted, while for $k \in \mathscr{I}_i^{\text{ne}}$ it is equal to the share of input $k$'s value out of the value of all inputs, i.e., its only exactly 1, if $i$ buys only one single non-essential input, $k$. Third, we can update the variable $h_i^{\text{d}}$ by evaluating the relative production function for each firm $i$

$$h_i^{\text{d}}(t+1) = \min\left[\min_{k\in\mathscr{I}_i^{\text{es}}}\left(\tilde{\Pi}_{ik}(t)\right),\, \tilde{\beta}_i + \sum_{k\in\mathscr{I}_i^{\text{ne}}} \tilde{\Pi}_{ik}(t)\right] \quad . \tag{15}$$

Note that the quantity $\sum_{k\in\mathscr{I}_i^{\text{ne}}} \tilde{\Pi}_{ik}(t)$ is 0 if all inputs $k \in \mathscr{I}_i^{\text{ne}}$ are not available. In the case of a pure Leontief producer this is always zero and $\tilde{\beta}_i = 1$, while for the case of a pure linear producer this quantity is one at time $t = 0$ and $\tilde{\beta}_i = 0$.

**Upstream recursion.** We follow the same procedure for the upstream update equation based on Eq. (3). The production of company $i$ in response to demand reductions from its customers is

$$x_i^{\text{u}}(t+1) \quad = \quad \sum_{j=1}^{n} W_{ij} h_j^{u}(t)$$

and we divide both sides by $x_i(0)$ to get

$$
\begin{aligned}
h_i^u(t+1) &= \sum_{j=1}^{n} \frac{W_{ij}}{\sum_{l=1}^{n} W_{il}} h_j^{\mathrm{u}}(t) \\
h_i^u(t+1) &= \sum_{j=1}^{n} \Lambda_{ji}^{\mathrm{u}} h_j^{\mathrm{u}}(t) \quad .
\end{aligned} \tag{16}
$$

The upstream impact matrix is defined as

$$
\Lambda_{ji}^{\mathrm{u}} = \begin{cases} \frac{W_{ij}}{\sum_{l=1}^{n} W_{il}} & \text{if} \quad W_{ij} > 0 \quad , \\ 0 & \text{else} \qquad\qquad , \end{cases} \tag{17}
$$

and its elements, $\Lambda_{ji}^{\mathrm{u}}$, determine the impact of the failure of buyer $j$ on supplier $i$. $\Lambda_{ji}^{\mathrm{u}} = 1$ only occurs if $j$ is the only buyer of $i$. Note that in comparison to the definition of the downstream update equations, we assume that upstream shocks are independent on the production functions. We assume implicitly that firms keep the proportion of their inputs fixed when output is reduced or increased. Therefore, we ignore that some inputs are "fixed costs" and will or cannot be affected by upstream contagion. Further, we can not take into account contractual obligations, which enforce a supply transaction also in case the demand for the customers product is reduced.

**Incorporating exogenous shocks.** For analysing an exogenous shock we have to make the initial exogenous shock to every firm explicit in the updating equations Eq. (16) and Eq. (15). Simply iterating these equations after the initial failure of firm $j$, $x_j^{\mathrm{d}}(1) = x_j^{\mathrm{u}}(1) = 0 \implies h_j^{\mathrm{d}}(1) = h_j^{\mathrm{u}}(1) = 0$, leads to an instant recovery of the initially failed firm because all its suppliers and customers are not affected (yet). Note that if there is a loop (e.g. $W_{ij} > 0$ and $W_{ji} > 0$) there will be a shock fed back to the initially defaulting firm. In our model the initial failure is an abstract exogenous shock to firm $j$ that could constitute everything from the destruction of the business premises of a firm, by for example a fire, a government mandated closure in a pandemic, to a strike. Technically, such exogenous shocks affect either capital, labour, or both in the production function. As mentioned in the main text, we don't model capital and labour explicitly but simply define an exogenous constraint, $\psi_i \in [0,1]$, and set it to the fraction of production that is *still possible* after the initial shock. For example, $\psi_i = 0.8$ means that 80% of production is still possible after the initial shock. Both shocks to labour or capital can be implemented with the variable $\psi_i$. Equations (16) and (15) change in the following ways

$$
h_i^{\mathrm{d}}(t+1) = \min\left[\min_{k \in \mathscr{I}_i^{\mathrm{es}}} \left(\tilde{\Pi}_{ik}(t)\right), \, \tilde{\beta}_i + \sum_{k \in \mathscr{I}_i^{\mathrm{ne}}} \tilde{\Pi}_{ik}(t), \psi_i\right] \quad , \tag{18}
$$

$$
h_i^u(t+1) = \min\left[\sum_{j=1}^{n} \Lambda_{ji}^{\mathrm{u}} h_j^{\mathrm{u}}(t), \psi_i\right] \quad . \tag{19}
$$

Note that instead of introducing the parameter, $\psi_i$, one could also work with incremental updates, $h_i^{\mathrm{d}}(t) - h_i^{\mathrm{d}}(t-1)$, as e.g. in[27]. When firms receive an exogenous shock, $(1-\psi_i) < 1$ (not a 100% failure) upstream and downstream shocks are propagated on top of the initial shock $1-\psi_i$. However, when including $\psi_i$ directly into upstream and downstream updates Eqs. (18-19), the additionally received upstream and downstream shocks need to be larger than the initial shock, $1-\psi_i$, so that firm $i$ propagates them further. Note that a positive shock can be considered if $\psi_i > 1$. As in[14] the initial shock could be made time dependent $\psi_i(t)$ to also model potential recovery from the initial shock.

**Modelling supplier replaceability.** So far the replaceability of suppliers was ignored. It is conceivable that in practice it is totally unrealistic for many firms (suppliers) to be not replaceable at all, even on the short-term; see also Supplementary Section S5. A straight forward proxy for a firm's replaceability — that can be inferred from available data — is its market share. Intuitively, a company with a small market share (within a fine grained industry classification) will be easier to replace than one with a large market share. For example, the lack of inputs caused by a (temporary) failure of a firm with a 5% market share can be most likely compensated to a large degree by its competitors, as long as the produced goods are reasonably standardized. This degree of standardisation varies significantly and is ignored in our approach. On the other hand a temporary failure of a firm with a 50% market share is much more difficult to compensate by competitors, given that there are inventory and production capacity constraints.

Modelling the replaceability of suppliers explicitly would require that a firm, whose supplier defaulted creates a new link with one or more firms of the same industry classification. This link creation depends on the available inventory level,

geographical proximity, and available production capacity. To be meaningful, such a network rewiring model needs to be calibrated to appropriate data that is hard to access. Moreover, it becomes computationally significantly more expensive and involved. We therefore choose a simpler approach for calculating the systemic risk index for every firm. We model the replaceability based on firms' market shares with a simple linear factor. In particular, we calculate the replaceability factor at time $t$

$$\sigma_i(t) = \min\Big(\frac{s_i^{\text{out}}(0)}{\sum_{j=1}^{n} s_j^{\text{out}}(0) h_j^d(t) \delta_{p_j,p_i}}, 1\Big) \quad . \tag{20}$$

Over time, $\sigma_i(t)$ can only increase and $\sigma_j(t) \in [0,1]$. It is bounded from below by firms with zero market share. When other suppliers of the same product have reduced outputs too, the supplier becomes harder to replace. Note that since the supply of firm $i$ itself is also part of the total market (denominator), a market share higher than 50% can not be replaced anymore by competitors. This would imply a short-term doubling of their capacities. Note that $\Lambda_{ji}^d\big(1-h_j^{\text{d}}(t)\big)$ is the shock that $i$ receives from $j$ and since $\sigma_j(t) \in [0,1]$, small market shares dampen this shock substantially.

To integrate this factor into the recursion we have to adapt the update equation for the availability of relative inputs $\tilde{\Pi}_{ik}(t)$. For $k \in \mathscr{I}_i^{\text{es}}$ we now update

$$\tilde{\Pi}_{ik}(t) = 1 - \sum_{j=1}^{n} \sigma_j(t) \Lambda_{ji}^d \big(1 - h_j^{\text{d}}(t)\big)\, \delta_{p_j,k} \quad , \tag{21}$$

and for $k \in \mathscr{I}_i^{\text{ne}}$ we define an artificial product category $k'$ that is updated according to

$$\tilde{\Pi}_{ik'}(t) = 1 - \sum_{k \in \mathscr{I}_i^{\text{ne}}} \sum_{j=1}^{n} \sigma_j(t) \Lambda_{ji}^d \big(1 - h_j^{\text{d}}(t)\big)\, \delta_{p_j,k} \quad . \tag{22}$$

Note that if all $\sigma_j(t) = 1$ and all $h_j^{\text{d}}(t) = 0$ for all non-essential input suppliers ($p_j \in \mathscr{I}_i^{\text{ne}}$) the right hand side of Eq. (22) is equal to $\tilde{\beta}_i$ and we can write the downstream update more compactly as

$$h_i^{\text{d}}(t+1) = \min\Big[\min_{k \in \mathscr{I}_i^{\text{es}}} \Big(\tilde{\Pi}_{ik}(t)\Big),\, \tilde{\Pi}_{ik'}(t), \psi_i\Big] \quad . \tag{23}$$

The matrix $P$ can be adjusted accordingly.

**Simplifications.** For completeness, we specify the simplification steps for the first term in Eq. (10)

$$\begin{aligned}
\frac{1}{\alpha_k} \frac{1}{x_i(0)} \sum_{j=1}^{n} W_{ji} h_j^d(t) \delta_{k,p_j} &= \\
\frac{\sum_{l=1}^{n} W_{il}}{\sum_{\iota=1}^{n} W_{\iota i} \delta_{p_\iota,k}} \frac{1}{\sum_{l=1}^{n} W_{il}} \sum_{j=1}^{n} W_{ji} h_j^d(t) \delta_{k,p_j} &= \\
\sum_{j=1}^{n} \frac{W_{ji}}{\sum_{\iota=1}^{n} W_{\iota i} \delta_{p_\iota,k}} h_j^d(t) \delta_{k,p_j} &= \\
\sum_{j=1}^{n} \Lambda_{ji}^{d1} h_j^d(t) \delta_{k,p_j} & \quad .
\end{aligned} \tag{24}$$

and the second term in Eq. (10)

$$\begin{aligned}
\frac{1}{x_i(0)} \frac{1}{\alpha_i} \sum_{k \in \mathscr{I}_i^{\text{ne}}} \sum_{j=1}^{n} W_{ji} h_j^{\text{d}}(t) \delta_{p_j,k} &= \\
\frac{1}{\sum_{l=1}^{n} W_{il}} \frac{\sum_{l=1}^{n} W_{il}}{\sum_{j=1}^{n} W_{ji}} \sum_{k \in \mathscr{I}_i^{\text{ne}}} \sum_{j=1}^{n} W_{ji} h_j^{\text{d}}(t) \delta_{p_j,k} &= \\
\sum_{k \in \mathscr{I}_i^{\text{ne}}} \sum_{j=1}^{n} \frac{W_{ji}}{\sum_{j=1}^{n} W_{ji}} h_j^{\text{d}}(t) \delta_{p_j,k} &= \\
\sum_{k \in \mathscr{I}_i^{\text{ne}}} \sum_{j=1}^{n} \Lambda_{ji}^{d2} h_j^{\text{d}}(t) \delta_{p_j,k} & \quad .
\end{aligned} \tag{25}$$

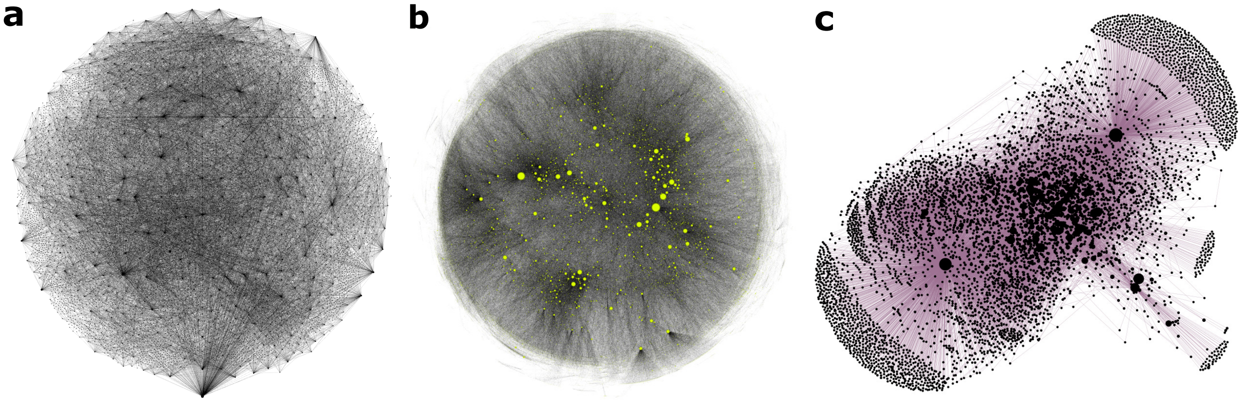


**Figure S2.** Visualizing different aspects of the production network. a) Section of the Hungarian production network with 5,541 nodes and 27,793 links. The network is obtained from considering the *same* 1,500 randomly sampled nodes as in Fig. 1 c) in the main text, considering all their suppliers, yielding 6,113 nodes. Here we consider *all links* between these 6,113 nodes and take the giant component from this. This shows that the true network is actually much denser. b) The supply links between all 91,595 firms in the Hungarian production network in 2017. Node size corresponds to strength. Large firms cluster in the center of the network. Darker areas correspond to denser parts. c) The induced subgraph of all neighbours from the largest three companies containing 5,945 firms and 26,783 links. It is clear that a large fraction the largest three nodes' buyers and suppliers are densely connected, while on the bottom and top right firms that are only direct neighbours of one out of the largest two firms are grouped together.

We denote the relative fraction of production that possible with only essential inputs $k \in \mathscr{I}_i^{\mathrm{es}}$ as

$$\tilde{\beta}_i = \frac{1}{x_i(0)}\beta_i = \frac{\sum_{k\in\mathscr{I}_i^{\mathrm{es}}}\sum_{j=1}^{n} W_{ji}\delta_{p_j,k}}{\sum_{j=1}^{n} W_{ji}\delta_{p_j,k}} \quad . \tag{26}$$

## S7 Description of Hungarian VAT data

To calibrate the production functions and to model the spreading of shocks, we use the supply network, $W$, consisting of all relevant supplier buyer transactions of firms within Hungary. For the current study we obtained data for the years 2017 and 2016. The firm level supplier-buyer connection data is collected by the National Tax and Customs Administration of Hungary as a part of the VAT reporting of firms. We got access to this dataset in an anonymized format through the Central Bank of Hungary. The data contains trade links among Hungarian companies between 2014 and 2017, when the tax content of the transactions between two firms exceeds HUF 1 million (EUR 3,000) in the given year. The cleaning and filtering of the data was based on[28]. The two most important corrections are described below.

Many of the links in the network disappear between the observed periods and new links emerge to a similar extent. This happens mainly because of the presence of many one-off, incidental transactions, which relationships are not particularly relevant from the point of view of supply chain contagion. As these links increase the noise in the data, we filtered the network to contain only long-term supplier connections. We consider a link long-term if there were at least two trade events between the parties and if there is at least 90 days time difference between the first and the last transaction. Under these mild requirements, only 54% of the links are long-term, however, these cover 93% of the aggregate trade volume in the network (in 2017).

In the Hungarian VAT regulation there is no general rule that states if firms belonging to the same ownership based group should report individually or at the group level. To handle the potential distortions arising from this inconsistency, we collapsed the supplier network to the group level in every case based on the ownership data obtained from the OPTEN database. The details of this procedure are described in[28].

To obtain not only the transactions between nodes, but also information about the nodes the VAT data set was enriched with anonymized firm level information from the Hungarian corporate tax dataset. This contains the NACE classification on the 4 digit level. By imputing NACE classifications from 2016 we can increase the number of firms to 69,863 or 76%. for 64,053 firms (in 2017) as well as revenue and material cost information. For those firms where no NACE classification is available, we merge it into an artificial 569$^{\text{th}}$ category. In the future, classification methods could be used to predict NACE membership based on the input and output links of a company.

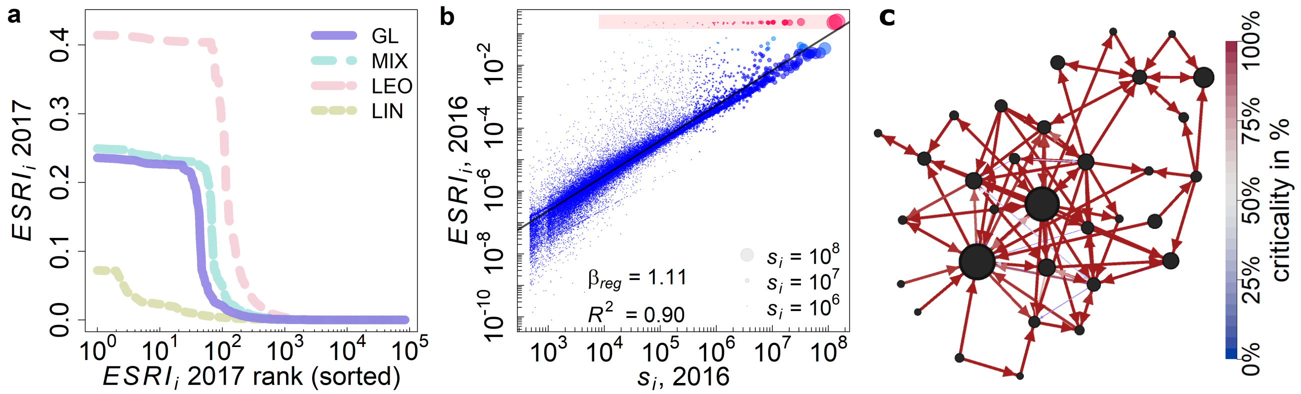


**Figure S3.** Economic systemic risk of companies. a) Economic systemic risk profile (distribution) $\mathrm{ESRI}_i$ for all $n = 85,131$ companies in linear-log scale for the year 2016. The distributions are rank-ordered, meaning the most risky company is to the very left. The blue line shows the result for the GL scenario (production functions according to industry classification and classifying produced goods as essential or not). A plateau exists around an ESRI of 0.22, containing 33 firms for GL. There is a steep decline to $\mathrm{ESRI}_i \sim 0.05$ from rank 33 to 57. 154 firms have an ESRI larger than 0.01. For comparison, the MIX scenario (light blue) is shown (production functions according to industry classification only). As limiting cases we show the LIN scenario (green), where all firms have linear production functions (lower bound) and for the upper bound, the situation where all firms are assumed to have Leontief production function (red). The tail of the ESRI profile decays as an approximate power-law; for the exponents see Fig. S4. b) ESRI plotted against firm strength (representing firm-size) in log-log scale. Symbol-size corresponds to the strength $s_i$, red symbols belong to the plateau, emphasised by the shaded area. We find large and small companies in the plateau, suggesting that firm-size is not a good predictor for very high ESRI. Even though we find that for the bulk of the companies strength explains parts of the variations in ESRI ($R^2 = 0.90$ and slope $\beta_{reg} = 1.11$ for regressing log-ESRI on log-strength, $p = 2 \cdot 10^{-16}$), for individual companies strength does not serve as a reliable predictor of ESRI. c) Network of the 32 most systemically risky firms (plateau) for 2016. Node size is proportional to the square root of strength, $s_i$. Link (from $i$ to $j$) colors correspond to the downstream "criticality" i.e. the percentage of $j$'s production should $i$ stop producing, $\Lambda^d_{ij}$. Red thick (blue, thin) links indicate very large (small) losses of production. Small companies predominately supply to large high-risk companies, thereby inheriting systemic risk. Between the companies in the plateau, almost all supply relations are highly critical (red thick). In this sub-network the default of one firm's production will lead to the default of many others.

## S8 Network visualisation

To give a more detailed view on the network structure, we show three additional aspects of it in Fig. S2. a) shows a section of the network with 5,541 nodes and all 27,793 links between them. It is based on the same randomly sampled 1,500 nodes used for the visualization in Fig. 1 c) in the main text. In Fig. 1 c) we sample 1,500 random nodes and consider all their *in*-links, yielding 6,113 nodes, and visualizing the giant component consisting of 4070 nodes. Here we consider *all* links between the 6,113 nodes —not just the in-links of the 1,500 nodes— and visualize the resulting giant component consisting of 5,541 nodes. The giant component becomes substantially larger, and needless to say, denser. This makes the structure more difficult to visualize. The center of the network is denser than the periphery. However, by considering all links, some nodes on the periphery have higher degrees (nodes with high degree on the outer part of the circle). This is most likely due to the nature of considering a random sample. In the overall network they are most likely not in the periphery, but more central. In Fig. S2 b) we show the giant component of 86,470 nodes and 227,355 links. The core-periphery structure is still visible with some denser regions (darker areas in the center and around); the network becomes substantially sparser towards the periphery. In the center large nodes, highlighed in yellow, are present. Size is proportional to the square root of node strength. This hints to the fact that denser regions are agglomerations around large firms and clusters of large firms. This should be verified with community detection methods in future work. In Fig. S2 c) we visualize the graph of all suppliers of the largest 3 nodes (5,945 nodes) and all 26,783 links between them. It is obvious that many suppliers of the largest firms are densely connected.

## S9 2016 results for ESRI

We show the same results as in main text Fig. 3 a), b) and c) also for the year 2016. The rank-ordered distribution of the ESRI is shown in Fig. S3 a) in log-linear scale for 2016. For the realistic baseline scenario GL (blue), we find that 32 companies show extremely high levels of systemic risk, all being at a value of about 0.22, meaning that about 22% of the entire economy

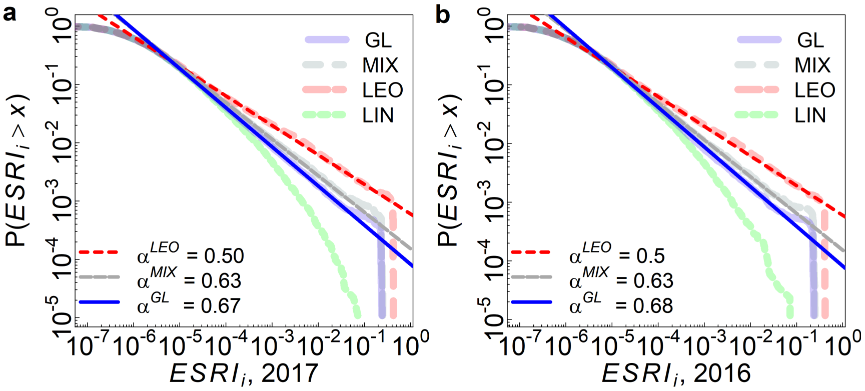


**Figure S4.** Power laws of ESRI distributions. Cumulative distribution of ESRI indicates a power law decay in the tail of the three scenarios: generalized Leontief (GL), mixed Leontief and linear (MIX), linear-only (LIN), and Leontief-only (LEO). a) values for the year 2017. The estimated exponents are 0.67 for GL, 0.63 for MIX, and 0.5 for LEO. b) shows the values for the year 2016 with the exponents 0.68 for GL, 0.63 for MIX, and 0.5 for LEO.

is affected should one of these companies fail and its supply and demand is not replaced. 57 and 154 firms have an ESRI larger than 0.05 and 0.01, respectively. For respective numbers for the other scenarios, see Tab. S2 in Supplementary Section S10. The situation is similar for the LL scenario, where 51 companies belong to the plateau with values around 0.22. For the (unrealistic) reference case, LEO, where *all* companies are of Leontief type (red), we find much higher systemic risk levels for 66 firms, with an ESRI of about 0.41.

For ranks larger than a characteristic rank of 32, the pronounced plateau in the distribution is followed first by a steep decline and then by a slow decay of the ESRI values. The tail (without the plateau and steep decline) of the distribution can be fitted to a power-law with an exponent of roughly $\alpha^{\mathrm{GL}} = 0.68$ for GL, and $\alpha^{\mathrm{MIX}} = 0.63$ for MIX. For details of the power-laws fit, see Fig. S4 b) in Supplementary Section S10. The shape of the rank-ordered ESRI distribution (plateau and power-law tail) is similar to what was found in Fig. 4. in[13]. As expected, the reference case LIN (green) where *all* companies have linear production functions, generates substantially lower systemic risk than GL and MIX. The LIN case does neither show a plateau nor a power-law decay.

To better understand which companies are forming the plateau region of extremely risky companies in Supplementary Section Fig. S3 b) we show $\mathrm{ESRI}_i$ as a function of the strength, $s_i$ (firm size within the network). Red color indicates the plateau companies; Symbol size represents the strength, $s_i$. Clearly, as for the year 2017, also in 2016 the ESRI of plateau firms (located in the shaded area) is not changing with size; in the plateau we find large and small companies (note the range of strength of 4 orders of magnitude), suggesting that firm-size is not able to predict extreme ESRI values at all also for the year 2016. For the bulk of the companies (blue), we find an overall strong correlation of log-ESRI and log-strength ($R^2 = 0.90$ and slope $\beta_{reg} = 1.11$ for regressing log-ESRI on log-strength, $p = 2 \cdot 10^{-16}$). For individual companies, strength does not serve as a reliable predictor of the ESRI, since the spread of the ESRI extends over 4 orders of magnitude. Note that all firms in the plateau are physical producers (NACE A01-F43).

Figure S3 c) shows the network between the plateau nodes. The pattern is similar to 2017. There is a strongly connected component of core nodes that are connected with each other via highly critical supply relationships. In the periphery there are mostly small nodes having only one or two critical supply relations to nodes in the strongly connected component.

## S10 Estimation of power-law exponents

We investigate the empirical distribution of the ESRI for the years 2017 and 2016 in more detail. In Fig. S4 a) we plot the empirical cumulative distribution function — indicating the probability $\mathrm{P}(\mathrm{ESRI}_i > x)$ of observing an ESRI value of larger than $x$ — in log-log scale, for the four scenarios, GL, MIX, LIN, and LEO. The three scenarios including shares of Leontief production functions behave differently than the LIN scenario. These three scenarios decay over a wide range of values as a power law (linearly in log-log), followed by a sharp cut-off (the plateau). The linear-only (LIN) scenario does not show this behaviour. We estimate the power-law exponents for the three scenarios and indicate the corresponding slopes with a red dashed line for LEO, a grey long-dashed line for MIX and a blue solid line for the GL. For the estimation we use the maximum likelihood estimator (MLE)

$$\hat{\alpha} = 1 + n \Big[ \sum_{i=1}^{n} \ln \Big( \frac{x_i}{x_{\min}} \Big) \Big]^{-1} \quad , \tag{27}$$

see[38] or Eq. (3.1) in[39]. Since, the linear decay does not extent over the whole range of the ESRI values we restrict the estimation intervals. For LEO the estimate gives a slope $\hat{\alpha}^{\mathrm{LEO}} = 0.5$ for the values $\mathrm{ESRI}_i \in [1.5 \cdot 10^{-6}, 10^{-1}]$ covering 53% of the observations. For the MIX the estimate gives a slope $\hat{\alpha}^{\mathrm{MIX}} = 0.63$ for the values $\mathrm{ESRI}_i \in [5 \cdot 10^{-6}, 3 \cdot 10^{-2}]$, covering 29% of the observations. For the GL the estimate gives a slope of $\hat{\alpha}^{\mathrm{GL}} = 0.67$ for the values $\mathrm{ESRI}_i \in [7 \cdot 10^{-6}, 3 \cdot 10^{-2}]$, covering 24% of the observations. The MLE in Eq. (27) corresponds to the probability density function, while the plotted empirical ESRI distributions and the estimated slopes $\hat{\alpha}^{\mathrm{GL}}, \hat{\alpha}^{\mathrm{MIX}}, \hat{\alpha}^{\mathrm{LEO}}$ in the figure corresponds to the cumulative distribution function that has an exponent $\alpha - 1$. Figure S4 b) shows the results for the year 2016. The patterns are practically identical. For GL the estimate of the slope changes slightly from 0.67 to 0.68.

Table S1 contains the number of observations above several threshold values of ESRI in 2017. The value 0.41 is the threshold for the plateau in the LEO scenario (66 firms) and 0.22 is the threshold for the plateau in GL (32 firms), and MIX (47 firms). In all scenarios only a few firms have a high ESRI of more than one percent. Same for 2016 in Table S2.

**Table S1.** Number of Large Observations 2017

| ESRI Levels | 0.41 | 0.22 | 0.1 | 0.05 | $10^{-2}$ | $10^{-3}$ | $10^{-4}$ |
|---|---|---|---|---|---|---|---|
| LIN | 0 | 0 | 0 | 2 | 32 | 431 | 3167 |
| GL | 0 | 32 | 50 | 63 | 165 | 784 | 3740 |
| MIX | 0 | 47 | 77 | 101 | 258 | 1041 | 4178 |
| LEO | 66 | 128 | 176 | 241 | 611 | 1880 | 5526 |

**Table S2.** Number of Large Observations 2016

| ESRI Levels | 0.4 | 0.21 | 0.1 | 0.05 | $10^{-2}$ | $10^{-3}$ | $10^{-4}$ |
|---|---|---|---|---|---|---|---|
| LIN | 0 | 0 | 0 | 2 | 33 | 392 | 3052 |
| GL | 0 | 32 | 45 | 57 | 154 | 687 | 3540 |
| MIX | 0 | 56 | 70 | 99 | 246 | 926 | 3984 |
| LEO | 66 | 113 | 163 | 231 | 591 | 1765 | 5210 |

**Table S3.** NACE 2 category counts for top 32 ESRI firms (plateau) in 2016 and 2017.

| NACE 2 | Name | 2016 | 2017 |
|---|---|---|---|
| A1 | Crop and animal production, hunting and related service activities | 1 | 0 |
| B6 | Extraction of crude petroleum and natural gas | 1 | 0 |
| B9 | Mining support service activities | 0 | 1 |
| C11 | Manufacture of beverages | 2 | 2 |
| C13 | Manufacture of textiles | 1 | 1 |
| C14 | Manufacture of wearing apparel | 1 | 0 |
| C15 | Manufacture of leather and related products | 1 | 1 |
| C17 | Manufacture of paper and paper products | 1 | 0 |
| C18 | Printing and reproduction of recorded media | 1 | 1 |
| C19 | Manufacture of coke and refined petroleum products | 1 | 1 |
| C20 | Manufacture of chemicals and chemical products | 5 | 8 |
| C23 | Manufacture of other non-metallic mineral products | 2 | 2 |
| C24 | Manufacture of basic metals | 1 | 0 |
| C26 | Manufacture of computer, electronic and optical products | 1 | 1 |
| C27 | Manufacture of electrical equipment | 2 | 3 |
| C28 | Manufacture of machinery and equipment n.e.c. | 3 | 1 |
| C29 | Manufacture of motor vehicles, trailers and semi-trailers | 0 | 1 |
| C32 | Other manufacturing | 0 | 2 |
| C33 | Repair and installation of machinery and equipment | 0 | 1 |
| D35 | Electricity, gas, steam and air conditioning supply | 6 | 5 |
| E36 | Water collection, treatment and supply | 1 | 1 |
| E39 | Remediation activities and other waste management services | 1 | 0 |

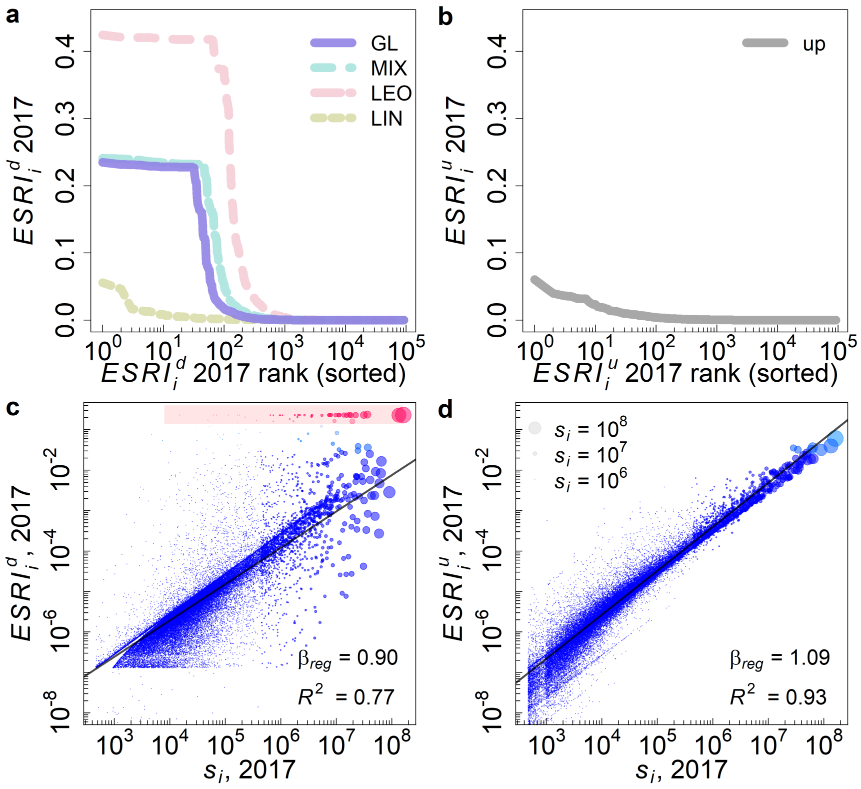


**Figure S5.** Downstream and upstream components of economic systemic risk. a) Downstream economic systemic risk profile (distribution) $\mathrm{ESRI}_i^{\mathrm{d}}$ of $n = 91,595$ companies in linear-log scale for 2017. Distributions are rank-ordered, meaning the most risky company is to the very left. The blue line shows the result for the realistic GL scenario, the MIX scenario is shown in light blue, the scenario LIN in green and the scenario LEO in red. b) Upstream economic systemic risk profile (distribution) $\mathrm{ESRI}_i^{\mathrm{u}}$ of $n = 91,595$ companies in linear-log scale for 2017. Note that the upstream economic systemic risk is the same across the four scenarios. c) GL scenario $\mathrm{ESRI}^{\mathrm{d}}$ plotted against firm strength (firm-size) in log-log scale. Symbol size represents strength $s_i$, red symbols belong to the plateau, emphasised by the shaded area. We find large and small companies in the plateau, suggesting that very high $\mathrm{ESRI}_i^{\mathrm{d}}$ is not determined by size. d) GL scenario $\mathrm{ESRI}_i^{\mathrm{u}}$ plotted against firm strength in log-log scale. Firm size explains upstream systemic risk better, even though for a given firm size there are considerable variations in $\mathrm{ESRI}_i^{\mathrm{u}}$.

## S11 Comparison of downstream and upstream shock propagation effects propagation

As pointed out in the main text, the quantity $\mathrm{ESRI}_i$ considers both systemic risk from downstream and upstream shock propagation. However, a priori it is not clear how strongly the two effects contribute to $\mathrm{ESRI}_i$. Therefore, we calculate the downstream economic systemic risk index, $\mathrm{ESRI}_i^{\mathrm{d}}$, by only considering the production losses firms incur from downstream shocks and the upstream economic systemic risk index $\mathrm{ESRI}_i^{\mathrm{u}}$, by only considering the production losses firms incur from upstream shocks. The two quantities are calculated by only considering firms production losses from the respective propagation direction, i.e.

$$\mathrm{ESRI}_j^{\mathrm{d}} = \sum_{i=1}^{n} \frac{s_i^{\mathrm{out}}}{\sum_{l=1}^{n} s_l^{\mathrm{out}}} \left(1 - h_i^{\mathrm{d}}(T)\right) \quad , \quad \mathrm{ESRI}_j^{\mathrm{u}} = \sum_{i=1}^{n} \frac{s_i^{\mathrm{out}}}{\sum_{l=1}^{n} s_l^{\mathrm{out}}} \left(1 - h_i^{\mathrm{u}}(T)\right) \quad . \tag{28}$$

Note that firms with no out-strength, $s_i^{\mathrm{out}}$, have no downstream systemic risk, $\mathrm{ESRI}_i^{\mathrm{d}} = 0$, because they can not affect any nodes downstream of them. However, firms having no in-strength, $s_i^{\mathrm{in}}$, will still have a slightly positive upstream systemic risk index, $\mathrm{ESRI}_i^{\mathrm{u}} = \frac{s_i^{\mathrm{out}}}{\sum_{l=1}^{n} s_l^{\mathrm{out}}}$, because we count also the weight of the initially failing firm $i$. We chose the weighting scheme with $s_i^{\mathrm{out}}$ deliberately, such that we can interpret ESRI intuitively as fraction of affected output in the production network. Furthermore, we do not deduct the weight of the initially failing firm, because we want ESRI to reflect the overall impact of a firm.

First, we show the rank-ordered distributions of $\mathrm{ESRI}^{\mathrm{d}}$ in Fig. S5 a). We see that in general the rank-ordered distributions are very similar to the ESRI distributions Fig. 3 a, however, there are a few noticeable differences. First, the plateau of the GL scenario is slightly flatter. The maximum ESRI is 0.242 and the maximum $\mathrm{ESRI}^{\mathrm{d}}$ is 0.235. The rank 32 ESRI is 0.227

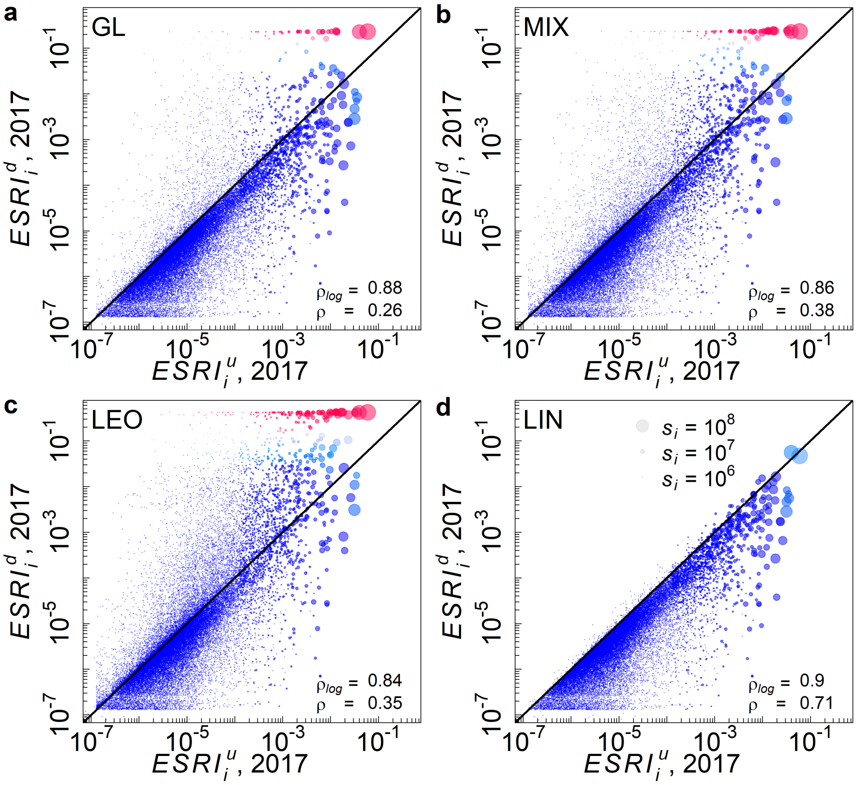


**Figure S6.** Downstream versus upstream economic systemic risk of firms in log-log scale for the four different scenarios GL, MIX, LEO, LIN. The x-axis shows the $\text{ESRI}_i^{\text{u}}$; the y-axis shows the $\text{ESRI}_i^{\text{d}}$. Diagonal identity lines are black ($\text{ESRI}_i^{\text{u}} = \text{ESRI}_i^{\text{d}}$). Bubble size is proportional to the square root of firm size; red color indicates high ESRI values, blue color low ESRI values. 21,771 are omitted in the log-log plot ($\text{ESRI}_i^{\text{d}} = 0$) and correlations of the log-ed variables. p-values are close to zero ($p = 2.2^{-16}$) for all correlation coefficients. a) GL scenario, most firms have higher upstream systemic risk, $\text{ESRI}_i^{\text{u}}$, than downstream systemic risk, $\text{ESRI}_i^{\text{d}}$, i.e. most bubbles are to the right of the black line. This is explained by the supplier replaceability factor dampening downstream shock propagation. The correlation of the logged variables is high with $\rho_{\text{log}} = 0.88$, but the regular correlation is low with $\rho_{\text{log}} = 0.26$. For the firms with the highest systemic risk (ESRI), downstream effects clearly dominate. b) MIX scenario, similar behaviour as the GL scenario, but more firms have higher downstream than upstream systemic risk. c) LEO scenario, a considerable part of firms have higher downstream than upstream systemic risk. For the bulk of low risk firms upstream systemic risk still larger dominates. d) LIN scenario, upstream systemic risk dominates for the vast majority of firms. Note that the correlations are highest for this scenario.

and the rank 32 $\text{ESRI}^{\text{d}}$ is 0.226. Similarly for the MIX scenario. Therefore, in the GL scenario upstream effects also play a role for firms with the highest risk, even though a comparably minor one. Intuitively, for the LEO scenario differences are even more negligible, because the non-linearities dominate stronger. For the LIN scenario we see that the maximum ESRI is 0.072. The maximum downstream $\text{ESRI}^{\text{d}}$ is substantially smaller with 0.056. $\text{ESRI}^{\text{d}}$ decays faster than ESRI, ranks 10 and 32 are 0.008 and 0.004 ($\text{ESRI}^{\text{d}}$) and 0.023 and 0.010 (ESRI), respectively. Fig. S5 b) shows the rank-ordered distribution for the upstream $\text{ESRI}^{\text{u}}$. In our model downstream and upstream shock propagation are non interacting and the specifications of the four considered scenarios affect only the downstream part. Hence, the upstream distribution is de facto the same for all four scenarios. The maximum $\text{ESRI}^{\text{u}}$ is 0.060 and ranks 10 and 31 are 0.023 and 0.009, respectively. Therefore, upstream effects are larger than downstream effects in the LIN scenario, but substantially smaller than in the other three scenarios. In Fig. S5 c) we plot downstream systemic risk, $\text{ESRI}^{\text{d}}$, against company size in log-log scale —analogously to Fig. 3 b). We see that both the slope, $\beta = 0.9$, and the coefficient of determination, $R^2 = 0.77$, are substantially lower than for ESRI, i.e. firm size preforms worse in explaining downstream systemic risk of a firm. In Fig. S5 d) we plot upstream systemic risk, $\text{ESRI}^{\text{u}}$, against company size in log-log scale. We see that the coefficient of determination, $R^2 = 0.93$, is substantially higher than for $\text{ESRI}^{\text{d}}$, and slightly higher for ESRI, i.e. firm size preforms better in explaining upstream systemic risk of firms. However, note that also upstream systemic risk, can vary orders of magnitude for a given firm size.

Next we directly compare the downstream and upstream systemic risk for the four different scenarios GL, MIX, LEO, LIN

in more detail. For each of the four scenarios Fig S6 a)-d) provides scatter plots in log-log scale with $\mathrm{ESRI}_i^{\mathrm{u}}$ on the x-axis and $\mathrm{ESRI}_i^{\mathrm{d}}$ on the y-axis. We summarize the most important observations. First, in all visually it seems that more firms have higher levels of upstream systemic risk than downstream systemic risk, i.e. lie to right of the diagonal. This effect is most present in the LIN scenario followed by the GL, MIX and LEO scenario. This can be explained by the fact that the supplier replaceability factor $\sigma$ dampens the downstream shock spreading and consequently the $\mathrm{ESRI}_i^{\mathrm{d}}$ values are lower. This effect is not present for the upstream shock propagation. Second, the non-linearity in the production functions for the GL, MIX and LEO scenarios counteracts this effect and leads to a considerable amount of firms having more downstream than upstream systemic risk. This effect is strongest for the LEO, scenario followed by MIX and GL. Third, the correlation between up- and downstream systemic risk in linear variables is high for LIN scenario ($\rho = 0.71$), but much lower for the other scenarios $\rho = 0.38$, $\rho = 0.35$, $\rho = 0.26$, for the MIX, LEO, GL scenarios, respectively. The correlations for the log systemic risk indices are considerably higher for all scenarios than the regular ones. Fourth, when non-linearities are present, firms with very high systemic risk are clearly dominated by downstream effects.

We analyze the economic systemic risk index, ESRI, reduction when ignoring upstream shock propagation for the GL and the LIN scenario in more detail. We calculate the relative ESRI reduction from as

$$\delta_i^{\mathrm{nu}} = \frac{\mathrm{ESRI}_i - \mathrm{ESRI}_i^{\mathrm{d}}}{\mathrm{ESRI_i}} \quad . \tag{29}$$

We calculate $\delta_i^{\mathrm{nu}}$ for the groups of firms with the following $\mathrm{ESRI}_i$ ranks in the GL scenario: 1 to 32 (plateau), 33 to 66 (steep decline), 64 to 165 ($\mathrm{ESRI}_i > 10^-2$), 166 to 784 ($\mathrm{ESRI}_i > 10^-3$) and 785 to 3740 ($\mathrm{ESRI}_i > 10^-24$). See also the second line in Table S1. For the GL scenario Table S4 shows that the mean ESRI reduction for the 32 plateau firms is small with 0.5%, but the reduction for the firm with highest ESRI would be a non negligible 5.8%. For the firms with rank 33 to 66 (steep decline) the reductions are negligible. For the ranks from 64 to 165 (firms with ESRI from 0.01 to 0.05) some firms are in this group already purely due to their upstream contagion, —without considering upstream contagion they would be systemically irrelevant— however the median reduction is still small with 0.6%; even though the mean reduction is 17.1%. For higher ranks the upstream component becomes more important. The median decrease when ignoring upstream contagion is 28% and 57% for the firms with ranks from 166 to 784, and 785 to 3740, respectively. These findings indicate that even in the GL scenario —exhibiting significant non-linearities in the production functions— upstream effects play a relevant role for overall economic systemic risk assessment.

**Table S4.** Relative ESRI reduction, $\delta_i^{\mathrm{nu}}$, when only considering $\mathrm{ESRI}^{\mathrm{d}}$ in the GL scenario.

| firm ranks | 1 to 32 | 33 to 63 | 64 to 165 | 166 to 784 | 785 to 3740 |
|---|---|---|---|---|---|
| Min. | 0.000 | 0.000 | 0.000 | 0.000 | 0.000 |
| $1^{\mathrm{st}}$ Qu. | 0.000 | 0.000 | 0.001 | 0.011 | 0.188 |
| Median | 0.001 | 0.000 | 0.006 | 0.282 | 0.568 |
| Mean | 0.005 | 0.001 | 0.171 | 0.402 | 0.525 |
| $3^{\mathrm{rd}}$ Qu. | 0.006 | 0.000 | 0.074 | 0.831 | 0.836 |
| Max. | 0.058 | 0.010 | 0.998 | 1.000 | 1.000 |

We show the relative reductions, $\delta_i^{\mathrm{nu}}$, for the same rank groups in the LIN scenario in Table S5. The results show that upstream contagion is the dominant force — mean and median reductions across all rank groups are larger than 50%— and, hence, confirm the intuition from Fig. S6.

**Table S5.** Relative ESRI reduction, $\delta_i^{\mathrm{nu}}$, when only considering $\mathrm{ESRI}^{\mathrm{d}}$ in the LIN scenario.

| firm ranks | 1 to 32 | 33 to 63 | 64 to 165 | 166 to 784 | 785 to 3740 |
|---|---|---|---|---|---|
| Min. | 0.040 | 0.000 | 0.000 | 0.000 | 0.000 |
| $1^{\mathrm{st}}$ Qu. | 0.475 | 0.703 | 0.448 | 0.422 | 0.394 |
| Median | 0.752 | 0.864 | 0.697 | 0.702 | 0.626 |
| Mean | 0.665 | 0.758 | 0.650 | 0.640 | 0.593 |
| $3^{\mathrm{rd}}$ Qu. | 0.915 | 0.975 | 0.950 | 0.916 | 0.856 |
| Max. | 0.998 | 1.000 | 1.000 | 1.000 | 1.000 |

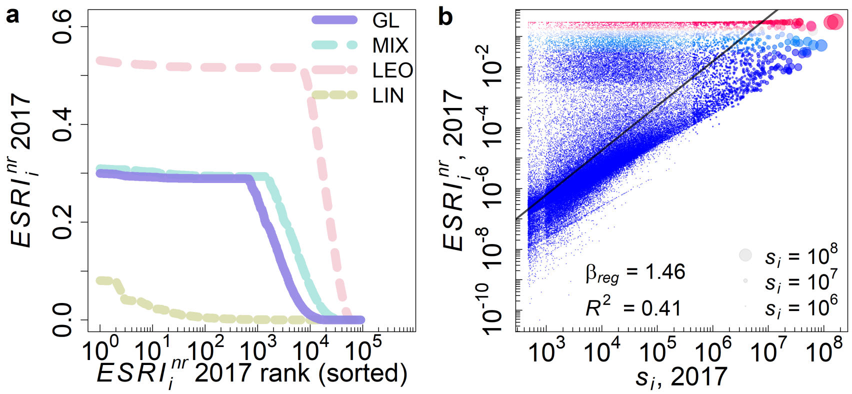


**Figure S7.** Economic systemic risk index without supplier replaceability factor $\sigma$. a) Economic systemic risk profile (distribution) $\text{ESRI}_i^{nr}$ without supplier replaceability factor for $n = 91,595$ companies in linear-log scale for 2017. Distributions are rank-ordered, meaning the most risky company is to the very left. The blue line shows the result for the realistic GL scenario, the MIX scenario is shown in light blue, the scenario LIN in green and the scenario LEO in red. Plateaus are higher for LEO, MIX and GL scenarios with maximum values of 0.53, 0.31 and 0.30, while containing 8079, 1565 and 743 firms, respectively. The maximal value for LIN is 0.08 and 35 firms have systemic risk larger than 0.01. b) GL scenario $\text{ESRI}^{rm}$ plotted against firm strength (firm-size) in log-log scale. Symbol size represents strength $s_i$, red symbols belong to the plateau. We find large and small companies in the plateau, suggesting that very high $\text{ESRI}_i^{\text{d}}$ is not determined by size. It is clearly visible that size does not correlate well with $\text{ESRI}^{rm}$.

## S12 Effect of the supplier replaceability factor on ESRI

We investigate the effect of introducing the supplier replaceability factor $\sigma$ (see Eq. (8) and Supplementary Section S5). The supplier replaceability factor only affects downstream shock propagation. We summarize the most relevant observations. Most strikingly, Fig. S12 a) shows that the plateaus of the three scenarios with non-linear production function components are substantially larger. They contain more firms, 8079, 1565 and 743 compared to 66, 47 and 32 for LEO, MIX and GL, respectively. The plateau heights increased from 0.41, 0.22 and 0.22 to 0.53, 0.31 and 0.30 for LEO, MIX and GL, respectively. The LIN scenario seems to be less affected with replaceability factor 32 firms had ESRI values higher than 0.01 compared to 35 without replaceability factor. Therefore, the supplier replaceability factor dampens shock propagation significantly. Fig. S12 b) shows that without the supplier replaceability factor ESRI becomes increasingly unrelated to firm size. The coefficient of determiniation drops to $R^2 = 0.41$ in the log-log regression. This is also visually apparent.

## S13 Regression Analysis

We conduct a more extensive regression analysis to investigate if there are firm level factors that explain $\text{ESRI}_i$ better than total strength, $s_i$. We estimate 5 additional regression models with the dependent variable in *log-scale*. Details of the model specification and results are shown in Table S6. Each column corresponds to one model fit. First, we regress systemic risk on log-in-strength, $\log s_i^{\text{in}}$, (first column) and on log-out-strength, $\log s_i^{\text{out}}$, (second column) separately and jointly (third column). Then, we regress log-systemic risk on log-total-strength (fourth column), $\log s_i$, and on log-revenue (fith column). Additionally, we control for the number of customers, the number of suppliers in the network, market shares of firms in their respective NACE 4 sector and add fixed effects for the NACE 4 industry affiliation of firms. Note that market share is important because it determines the degree by which firms can be replaced as a supplier. Note that for in-strength and out-strength not all observations can be included, because there are firms without in- or out-links in the production network dataset.

We see that all independent variables are highly significant, which is not surprising given the large number of observations. Note that log-out-strength seems to be a better explanatory variable ($R^2 = 0.82$) than log-in-strength ($R^2 = 0.73$) for log-ESRI. The likely reason is that due to the non-linearity of the downstream contagion (for firms with essential inputs) downstream effects weigh larger than upstream effects. Interestingly, explanatory power ($R^2 = 0.85$) increases only slightly when adding both log-in- and log-out-strength. The difference in sample size makes a direct comparisons difficult. Interestingly, the explanatory power of log-total-strength ($R^2 = 0.91$) seems to be a better explanatory variable than log-in-strength and log-out-strength jointly. This can be attributed to the difference in the observations used in the model estimation. Note that the model containing log-total-strength consists of all 91,515 observations while the model containing log-in- and log-out-strength contains 35,058 observations. The ESRI of firms having both in- and out-links seems to be more difficult to explain than the one for firms having only in- or out-links. Log-revenue can explain less than either of the other four models. This is not surprising, because

revenue captures also sales transactions that are not present in the Hungarian production network and this leads to a distorted picture of firm size within the network. For example, a firm with a large revenue can export almost its entire production and thus cause relatively small downstream contagion, while the firm can import most of its inputs and thus causes only little upstream contagion. The additional control variable market share and the industry fixed effects increased the $R^2$ only marginally.

There is a clear indication that size proxies like strength seem to explain large parts in the variation of ESRI. However, as seen in main text Fig. 3 b) on the individual firm level for a given level of strength there exists still an extremely large variation in ESRI.

**Table S6.** Regression results. For the description of the five models, see text.

| | *Dependent variable: Log of economic systemic risk index (ESRI) of firms* | | | | |
|---|---|---|---|---|---|
| | (1) | (2) | (3) | (4) | (5) |
| log(in_strength) | 1.084$^{***}$ | | 0.548$^{***}$ | | |
| | (0.003) | | (0.003) | | |
| log(out_strength) | | 0.997$^{***}$ | 0.513$^{***}$ | | |
| | | (0.002) | (0.003) | | |
| log(total_strength) | | | | 1.095$^{***}$ | |
| | | | | (0.001) | |
| log(revenue) | | | | | 0.943$^{***}$ |
| | | | | | (0.004) |
| market_share | 2.922$^{***}$ | 3.108$^{***}$ | 2.293$^{***}$ | 1.890$^{***}$ | 2.469$^{***}$ |
| | (0.110) | (0.076) | (0.069) | (0.054) | (0.126) |
| number_of_buyers | 0.005$^{***}$ | −0.002$^{***}$ | −0.00004 | 0.0003$^{**}$ | 0.007$^{***}$ |
| | (0.0003) | (0.0002) | (0.0002) | (0.0002) | (0.0003) |
| number_of_suppliers | −0.003$^{***}$ | 0.011$^{***}$ | 0.001$^{***}$ | −0.0002 | 0.011$^{***}$ |
| | (0.0003) | (0.0002) | (0.0002) | (0.0002) | (0.0004) |
| Constant | −24.656$^{***}$ | −23.353$^{***}$ | −23.951$^{***}$ | −25.311$^{***}$ | −24.006$^{***}$ |
| | (0.043) | (0.028) | (0.035) | (0.018) | (0.053) |
| Observations | 56,829 | 69,824 | 35,058 | 91,595 | 68,834 |
| $R^2$ | 0.731 | 0.819 | 0.854 | 0.908 | 0.550 |
| Adjusted $R^2$ | 0.730 | 0.819 | 0.854 | 0.908 | 0.550 |
| Residual Std. Error | 1.163 | 0.873 | 0.710 | 0.632 | 1.417 |
| | (df = 56738) | (df = 69733) | (df = 34966) | (df = 91504) | (df = 68745) |
| F Statistic | 1,709.566$^{***}$ | 3,500.626$^{***}$ | 2,256.226$^{***}$ | 10,061.870$^{***}$ | 956.684$^{***}$ |
| | (df = 90; 56738) | (df = 90; 69733) | (df = 91; 34966) | (df = 90; 91504) | (df = 88; 68745) |

All models are estimated with NACE 4 industry fixed effects. $^{*}$p<0.1; $^{**}$p<0.05; $^{***}$p<0.01

## S14 Change of ESRI from 2016 to 2017

We investigate the changes of the systemic risk index, $\text{ESRI}_i$, and firm strength, $s_i$ from 2016 to 2017. For a comprehensive view we present respective bi-variate density plots in log-log scale in Fig. S8 a) and b). It is clear that most values lie on the diagonal indicated by red and dark blue colors. There are also large outliers visible (yellow). Note that these are single counts. The most notable outliers are constituted by the fluctuations in the plateau, i.e. nodes entering and leaving the plateau from 2016 to 2017. In general, large fluctuations are more likely for smaller values of ESRI while for larger values the spread away from the diagonal becomes smaller. For completeness we checked these relations by computing the

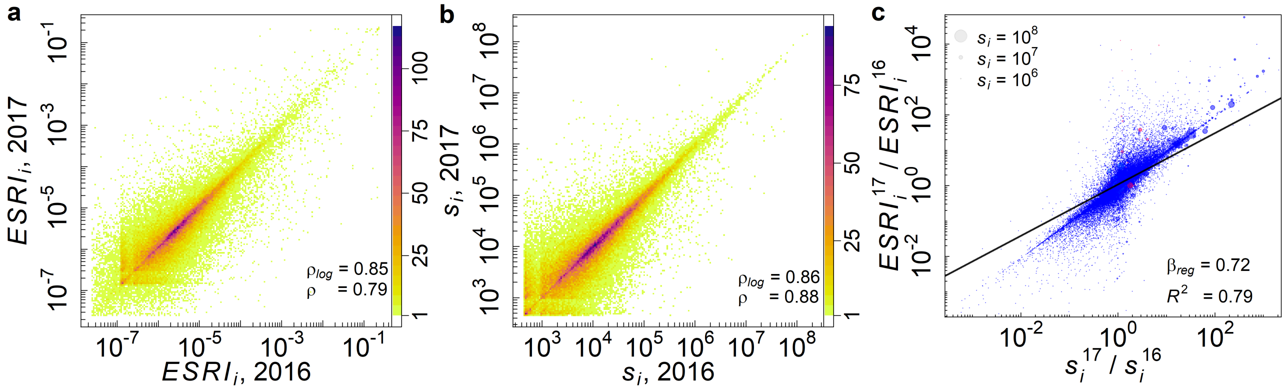


**Figure S8.** Temporal changes from 2016 to 2017. a) bivariate density plot of ESRI in 2016 versus 2017 in log-log. Yellow indicates low, blue high counts. The distribution is strongly peaked along the diagonal (red and blue). Note that the 1% of firms with the smallest ESRI values was omitted for the figure. b) bivariate density of firm strength $s_i$ 2016 versus $s_i$ 2017. The strength of smaller firms shows much stronger fluctuations than larger ones. The smallest 0.3% of firms was omitted in the figure. c) relative changes of ESRI plotted against relative changes of firm strength in log-log scale from 2016 to 2017. There are large relative changes in both strength, $s_i$ and ESRI$_i$. The regression for the logged variables gives $R^2 = 0.79$ and a slope of $\beta_{\text{reg}} = 0.72$ for a p-value of $p = 2 \cdot 10^{-16}$. Thus, the variation in firm strength fails to explain a large fraction of variation in firms' systemic risk, ESRI. Note the very large fluctuations in ESRI for the region where almost no change in strength occurred. On a broader scale, two variables are positively related left-bottom and right-top. For the bulk of values the fluctuations are relatively small.

correlation of the ESRI values of 2016 vs. 2017 in linear scale $\rho\left(ESRI^{16}, ESRI^{17}\right) = 0.78$ (p-value $2\,10^{-16}$), and in log-log scale $\rho\left(\log(ESRI^{16}), \log(ESRI^{17})\right) = 0.85$ (p-value $2\,10^{-16}$). The high correlation values confirm the results seen by visual inspection.

The relation of the most important firm-size proxy, firm strength $s_i$, from 2016 to 2017 is illustrated in Fig. S8 b). Again most values are found on the diagonal indicated by red and dark blue colors. Outliers are visible (yellow). Strength of smaller firms shows much larger fluctuations than the strength of of larger firms, seen by a diminishing variance around the diagonal for large values. The correlation of firm strength of 2016 and 2017 in linear scale is $\rho\left(ESRI^{16}, ESRI^{17}\right) = 0.86$ (p-value $2\,10^{-16}$) and correlation in log scale is $\rho\left(\log(ESRI^{16}), \log(ESRI^{17})\right) = 0.88$ (p-value $2\,10^{-16}$). This confirms the intuition obtained from the visual inspection.

Figure S8 c) shows the relative changes in firm level systemic risk $\left(\text{ESRI}_i^{17}/\text{ESRI}_i^{16}\right)$ against relative changes in strength $\left(\text{s}_i^{17}/\text{s}_i^{16}\right)$ in log-log scale. There are three main observations. First, the bulk of values is clustered at the center with small changes in both variables. Second, there is a group of firms exhibiting a positive relation between changes in strength and ESRI. Third, there is a group of firms exhibiting large variation in ESRI, but almost no variation in strength. Overall this indicates again that changes in strength seems to have some influence on changes in ESRI, but for many firms the changes in strength are not predictive for changes in ESRI. It seems that it matters more with whom firms form buyer-supplier relations than how large the sum of these relations is. For completeness we estimate the regression in log-log scale and receive an $R^2 = 0.79$ indicating that variations in relative change of strength explain 79% of the variations in relative change of ESRI. The slope is $\beta_{\text{reg}} = 0.72$. It is obvious from Fig. S8 c) that the regression line (black line) is not a good model for the data.

## S15 On the necessity of working at the firm level

Here we explain deeper why sector level aggregation of the firm level production network leads to distortions in the picture of shock propagation. If a sector contains at least two firms, A and B, with linearly independent input sector vectors or customer sector vectors, it is possible to construct three firm level shock scenarios that have the same size on the sector level, but are in fact heterogeneous shocks on the firm level (single companies are affect differently). Consequently, these three shock scenarios affect the direct input- and customer-sectors in different ways and hence actual shocks will propagate very differently for each of the scenarios. Note that shocks only propagate differently when analyzed on the firm level, but when the production network is aggregated to the sector level the cascades triggered by these three initial shocks are the same.

We present a concrete example where three shock scenarios are indistinguishable in terms of sector size affected, but have very different effects on how other sectors are affected by them. The implied assumption of a classical sector shock of size $x$% is that all companies $i$ within the sector are affected by the same percentage shock of $x_i\% = x\%$. In our example the

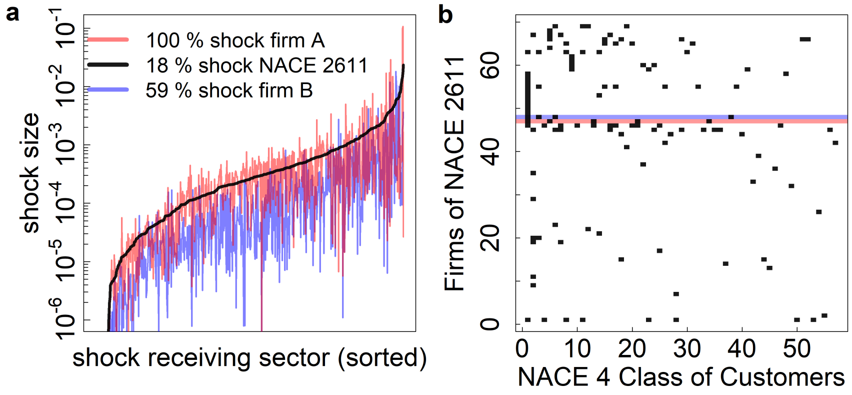


**Figure S9.** Firm level vs. sector level shocks. a) Effects of different shock scenarios to NACE sector 2611 (Manufacture of electronic components). Shocks are of the same size but affect different companies in the other 567 NACE 4 sectors. The black line shows the baseline shock scenario where a 18% shock is applied to all companies in sector 2611. Sectors are sorted in ascending order w.r.t. the baseline scenario. The red curve corresponds to a 100% shock to firm A (corresponds to an 18% shock on sector). The blue curve represents a 59% shock to firm B (corresponds to an 18% shock on sector). so that the three shock scenarios are all of the same size on the sector level. The choice of the initially shocked companies have a drastically different effect on how all the other sectors are affected. b) Customer sector vectors (at NACE 4 level) for 69 firms in sector 2611. Even though all companies belong to the same sector, their customer sector vectors are substantially different. We highlight the customer sectors of firm A (red, 5 distinct customer sectors) and firm B (blue highlight, 4 customer sectors). They differ substantially and have only 1 common customer sector.

sector shock is the baseline scenario. We simulate a 18% shock to each company in NACE sector 2611 (Manufacturing of electronic components). The percentage shock is measured in percent of the sector's strength, $s^{2611} = \sum_{j:p_j=2611} s_j$, that is initially affected. Then, we construct two firm level shocks that affect also 18% of the strength $s^{2611}$ of sector 2611, but are not uniformly distributed across the sector's firms. In the first scenario we apply a shock to a single company —called A for anonymity reasons— of 100% (de facto a temporary failure). Note that $s_A/s^{2611} = 0.18$. In the same way we apply a shock of 59% to firm B (we assume that 59% of the firms inputs and outputs are not bought and supplied). Note that $0.59 s_A/s^{2611} = 0.18$. Thus all three shocks are the same on the sector level in the classical sense, but affect different companies within the sector to a different degree. We know from Fig. S9 b) and main text Fig. 5 b) that firm A and B have different customer- and input-sector vectors, consequently, first order shocks spread to different sectors. This leads to a distinct spreading of shocks within the whole network and we expect other sectors to receive different indirect shocks for the three scenarios.

In Fig. S9 a) we show how all the other 567 NACE 4 sectors are affected, measured in percent of output lost, i.e., for each NACE sector (on the x-axis) the y-axis shows the percentage of the shock this NACE sector received in response to the initial shocks from the three scenarios uniform (black line) shock to firm A (red) and shock to firm B (blue). We sorted the NACE 4 sectors on the x-axis in ascending order with respect to the baseline scenario (the uniform sector shock), i.e., to the very left there is the NACE category that is affected least by the 18% shock to all firms in sector 2611. We see that the effects of the shock to firm A are in general larger than the shock to firm B, but this is not the case for every single sector. The blue line is below the baseline scenario in most cases (shock to firm A affects sectors less), while in several cases the red line is above the baseline scenario (shock to firm B affects these sectors stronger than the baseline). Note that the red (blue) line is above the black line only in 37% (6%) of the cases. This means that looking at the sector level would overestimate the shocks received by the NACE 4 sectors in 63% (94 %) of the cases and underestimate it in the other cases if the true shock was a shock to firm B (firm A). The correlation between the percentage of output lost per sector, caused by the initial shock scenarios, is given in Table S7. We see that the two firm level shocks have a low, but significant correlation of 0.11 (p-value $p = 0.0085$). Both firm level scenarios are positively correlated with the baseline shock scenario.

**Table S7.** Sector shock correlations

| shock scenario | shock 2611 | shock firm A | shock firm B |
|---:|---:|---:|---:|
| shock 2611 | 1.00 | 0.52 | 0.40 |
| shock firm A | 0.52 | 1.00 | 0.11 |
| shock firm B | 0.40 | 0.11 | 1.00 |

In Fig. S9 b) we show for all companies in NACE sector 2611 the customer sectors at the NACE 4-digit level (one row corresponds to one firm, a column to a NACE 4 class). Companies (rows) are sorted with respect to the similarity of their customer sectors. Even though all companies belong to the same fine grained industry sector, their customer sectors vary substantially. The customer sectors of firm A (red shading) and firm B (blue shading) have a small overlap seen in a Jaccard index of 0.13. Depending on which of two companies fail, very different customer sectors are affected. Among the 51 firms (in NACE 2611 that have NACE 4 classified inputs) 85% have no pairwise customer sector overlap (Jaccard index of zero). This means that when choosing two firms randomly within the sector the probability of having no common customer sector is 85%. Consequently, for theses cases not only different companies, but entirely different economic sectors will be affected, depending on which of the two randomly selected firms suffers the initial shock. The most common customer sector is supplied to by 30% of the companies in 2611 and the most common input sector is a supplier to 35% of the companies in 2611. Note that 19 firms have no input sectors (empty rows).

## References


1. Leontief, W. The economy as circular flow. *Archiv fur Sozialwissenschaft und Sozialpolitik* **60**, 577–623 (1928).
2. Leontief, W. The economy as a circular flow. *Struct. Chang. Econ. Dyn.* **2**, 181–212, DOI: https://doi.org/10.1016/0954-349X(91)90012-H (1991).
3. Miller, R. E. & Blair, P. D. *Input-Output Analysis: Foundations and Extensions* (Cambridge University Press, 2009).
4. Bak, P., Chen, K., Scheinkman, J. & Woodford, M. Aggregate fluctuations from independent sectoral shocks: self-organized criticality in a model of production and inventory dynamics. *Ricerche Econ.* **47**, 3–30 (1993).
5. Gabaix, X. The Granular Origins of Aggregate Fluctuations. *Econometrica* **79**, 733–772 (2011).
6. Acemoglu, D., Carvalho, V. M., Ozdaglar, A. & Tahbaz-Salehi, A. The Network Origins of Aggregate Fluctuations. *Econometrica* **80**, 1977–2016 (2012).
7. Carvalho, V. M. From Micro to Macro via Production Networks. *J. Econ. Perspectives* **28**, 23–48 (2014).
8. Hallegatte, S. An Adaptive Regional Input-Output Model and its Application to the Assessment of the Economic Cost of Katrina. *Risk Analysis: An Int. J.* **28**, 779–799 (2008).
9. Pichler, A., Pangallo, M., del Rio-Chanona, R. M., Lafond, F. & Farmer, J. D. Production Networks and Epidemic Spreading: How to Restart the UK Economy? (2020).
10. Colon, C., Hallegatte, S. & Rozenberg, J. Criticality analysis of a country's transport network via an agent-based supply chain model. *Nat. Sustain.* **4**, 209–215, DOI: https://doi.org/10.1038/s41893-020-00649-4 (2021).
11. Atalay, E., Hortacsu, A., Roberts, J. & Syverson, C. Network structure of production. *Proc. Natl. Acad. Sci.* **108**, 5199–5202, DOI: 10.1073/pnas.1015564108 (2011).
12. Magerman, G., De Bruyne, K., Dhyne, E. & Van Hove, J. Heterogeneous firms and the micro origins of aggregate fluctuations. Tech. Rep., NBB Working Paper (2016).
13. Moran, J. & Bouchaud, J.-P. May's instability in large economies. *Phys. Rev. E* **100**, 032307 (2019).
14. Inoue, H. & Todo, Y. Firm-level propagation of shocks through supply-chain networks. *Nat. Sustain.* **2**, 841–847 (2019).
15. Freixas, X., Parigi, B. M. & Rochet, J. C. Systemic Risk, Interbank Relations and Liquidity Provision by the Central Bank. *J. Money, Credit. Bank.* **32**, 611–638, DOI: 10.2307/2601198 (2000).
16. Boss, M., Elsinger, H., Summer, M. & 4, S. T. Network topology of the interbank market. *Quant. Finance* **4**, 677–684 (2004).
17. Eisenberg, L. & Noe, T. H. Systemic Risk in Financial Systems. *Manag. Sci.* **47**, 236–249, DOI: 10.1287/mnsc.47.2.236.9835 (2001).
18. Furfine, C. H. Interbank Exposures: Quantifying the Risk of Contagion. *J. Money, Credit. Bank.* **35**, 111–128 (2003).
19. Boss, M., Summer, M. & Thurner, S. Contagion Flow through Banking Networks. In *International Conference on Computational Science*, 1070–1077, DOI: 10.1007/978-3-540-24688-6_138 (Springer, 2004).
20. Elsinger, H., Lehar, A. & Summer, M. Risk Assessment for Banking Systems. *Manag. Sci.* **52**, 1301–1314 (2006).
21. Cont, R., Moussa, A. & Santos, E. Network Structure and Systemic Risk in Banking Systems. *SSRN, doi:10.2139/ssrn.1733528* DOI: 10.2139/ssrn.1733528 (2010).

**22.** Battiston, S., Puliga, M., Kaushik, R., Tasca, P. & Caldarelli, G. DebtRank: Too Central to Fail? Financial Networks, the FED and Systemic Risk. *Sci. Reports* **2** (2012).

**23.** Nier, E., Yang, J., Yorulmazer, T. & Alentorn, A. Network models and financial stability. *J. Econ. Dyn. Control.* **31**, 2033–2060, DOI: 10.1016/j.jedc.2007.01.014 (2007).

**24.** Gai, P. & Kapadia, S. Contagion in financial networks. *Proc. Royal Soc. A: Math. Phys. Eng. Sci.* **466**, 2401–2423, DOI: 10.1098/rspa.2009.0410 (2010).

**25.** Beale, N. *et al.* Individual versus systemic risk and the regulator's dilemma. *Proc. Natl. Acad. Sci.* **108**, 12647–12652, DOI: 10.1073/pnas.1105882108 (2011).

**26.** Arinaminpathy, N., Kapadia, S. & May, R. M. Size and complexity in model financial systems. *Proc. Natl. Acad. Sci.* **109**, 18338–18343, DOI: 10.1073/pnas.1213767109 (2012).

**27.** Bardoscia, M., Battiston, S., Caccioli, F. & Caldarelli, G. Debtrank: A Microscopic Foundation for Shock Propagation. *PLOS ONE* **10**, e0130406, DOI: 10.1371/journal.pone.0134888 (2015).

**28.** Borsos, A. & Stancsics, M. Unfolding the hidden structure of the Hungarian multi-layer firm network. Tech. Rep., Magyar Nemzeti Bank (Central Bank of Hungary) (2020).

**29.** EUROSTAT. Your companion guide to international statistical classifications. section iv - description of the main economic classifications (2021).

**30.** McFadden, D. Constant Elasticity of Substitution Production Functions. *The Rev. Econ. Stud.* **30**, 73–83 (1963).

**31.** Varian, H. R. *Intermediate Microeconomics: A Modern Approach: Ninth International Student Edition* (WW Norton & Company, 2014).

**32.** Douglas, P. H. The Cobb-Douglas Production Function Once Again: Its History, Its Testing, and Some New Empirical Values. *J. Polit. Econ.* **84**, 903–915, DOI: 10.1086/260489 (1976).

**33.** Carvalho, V. M. & Tahbaz-Salehi, A. Production Networks: A Primer. *Annu. Rev. Econ.* **11**, 635–663 (2019).

**34.** Wu, D. Essays on the interface between finance and technology (2016).

**35.** Rauch, J. E. Networks versus markets in international trade. *J. Int. Econ.* **48**, 7–35, DOI: https://doi.org/10.1016/S0022-1996(98)00009-9 (1999).

**36.** Giannetti, M., Burkart, M. & Ellingsen, T. What You Sell Is What You Lend? Explaining Trade Credit Contracts. *The Rev. Financial Stud.* **24**, 1261–1298, DOI: 10.1093/rfs/hhn096 (2011). https://academic.oup.com/rfs/article-pdf/24/4/1261/24441029/hhn096.pdf.

**37.** Barrot, J.-N. & Sauvagnat, J. Input Specificity and the Propagation of Idiosyncratic Shocks in Production Networks *. *The Q. J. Econ.* **131**, 1543–1592, DOI: 10.1093/qje/qjw018 (2016).

**38.** Hanel, R., Corominas-Murtra, B., Liu, B. & Thurner, S. Fitting power-laws in empirical data with estimators that work for all exponents. *PloS one* **12**, e0170920, DOI: https://doi.org/10.1371/journal.pone.0170920 (2017).

**39.** Clauset, A., Shalizi, C. R. & Newman, M. E. J. Power-Law Distributions in Empirical Data. *SIAM Rev.* **51**, 661–703, DOI: 10.1137/070710111 (2009).
